# Effect of Post-transplant Dietary Restriction on Hematopoietic Reconstitution and Maintenance of Reconstitution Capacity of Hematopoietic Stem Cells

**DOI:** 10.1007/s12015-024-10754-y

**Published:** 2024-07-05

**Authors:** Si Tao, Xingxing Qiu, Yiting Wang, Rongrong Qiu, Chenghui Yu, Man Sun, Lulu Liu, Zhendong Tao, Liu Zhang, Duozhuang Tang

**Affiliations:** 1 Department of Oncology, The Second Affiliated Hospital of Nanchang University, Jiangxi, China; 2 Department of Hematology, The Second Affiliated Hospital of Nanchang University, Jiangxi, China; 3 Jiangxi Key Laboratory of Clinical and Translational Cancer Research, Department of Oncology, The Second Affiliated Hospital of Nanchang University, Jiangxi, China; 4 Jiangxi Provincial Key Laboratory of Hematological Diseases (2024SSY06052), Department of Hematology, The Second Affiliated Hospital of Nanchang University, Min-De Road. 1, Nanchang City, 330006 Jiangxi Province China; 5 Department of Medical Oncology, First Affiliated Hospital, School of Medicine, Zhejiang University, Hangzhou, Zhejiang China; 6 Department of Medical Laboratory Medicine, Jiangxi Province Hospital of Integrated Chinese & Western Medicine, Jiangxi, China; 7 Intensive Care Unit, Beijing Jishuitan Hospital, Capital Medical University, Beijing, China

**Keywords:** Dietary restriction, Post-transplant, Hematopoiesis

## Abstract

Hematopoietic cell transplantation (HCT) is an important therapy for many hematological malignancies as well as some non-malignant diseases. Post-transplant hematopoiesis is affected by multiple factors, and the mechanisms of delayed post-transplant hematopoiesis remain poorly understood. Patients undergoing HCT often suffer from significantly reduced food intake due to complications induced by preconditioning treatments. Here, we used a dietary restriction (DR) mouse model to study the effect of post-transplant dietary reduction on hematopoiesis and hematopoietic stem cells (HSCs). We found that post-transplant DR significantly inhibited both lymphopoiesis and myelopoiesis in the primary recipient mice. However, when bone marrow cells (BMCs) from the primary recipient mice were serially transplanted into secondary and tertiary recipient mice, the HSCs derived from the primary recipient mice, which were exposed to post-transplant DR, exhibited a much higher reconstitution capacity. Transplantation experiments with purified HSCs showed that post-transplant DR greatly inhibited *hematopoietic stem cell (HSC)* expansion. Additionally, post-transplant DR reshaped the gut microbiotas of the recipient mice, which inhibited inflammatory responses and thus may have contributed to maintaining HSC function. Our findings may have important implications for clinical work because reduced food intake and problems with digestion and absorption are common in patients undergoing HCT.

**Graphical Abstract:**

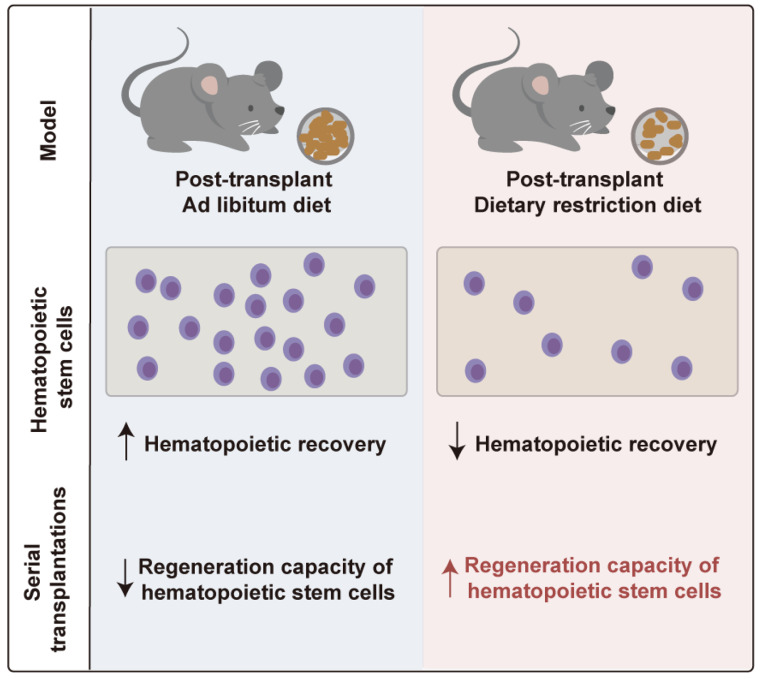

**Supplementary Information:**

The online version contains supplementary material available at 10.1007/s12015-024-10754-y.

## Introduction

Hematopoietic cell transplantation (HCT) offers curative potential for patients with hematological malignancies, red blood cell disorders, bone marrow failure, severe immune deficiency, and certain metabolic disorders [1, 2]. Early completion of post-transplant hematopoiesis can help reduce hospitalization costs and life-threatening complications such as infection and bleeding, thereby improving overall survival rates. Multiple factors, such as hematopoietic stem cell (HSC) functioning and the recipient’s environment, affect post-transplant hematopoiesis [1, 3–5]. Previous studies have shown that aging of *hematopoietic stem cells (HSCs)* negatively regulates their homing efficiency and long-term reconstitution capacity [3, 6]. Furthermore, transplanted HSCs require support from the bone marrow microenvironment, known as the niche, and the systemic environment of the recipients [1, 2]. Mouse models have shown that *gut microbiota* depletion can decrease energy harvesting and impair hematopoiesis after bone marrow transplantation [2, 7]. However, the mechanisms of delayed post-transplant hematopoiesis remain poorly understood.

We previously reported that in mouse models of homeostasis, a dietary restriction (DR) of a 30% reduction in daily food intake can reverse the differentiation tendency of HSCs, causing them to increase myeloid differentiation while inhibiting lymphoid differentiation, thereby inhibiting lymphoid hematopoiesis [8]. Long-term DR (6–9 months) can enhance the hematopoietic reconstitution function of aged HSCs [8, 9]. However, in transplantation models, HSCs are pressured to rebuild the entire hematopoietic system. This pressure forces the HSCs that are in a deep quiescent state under steady-state conditions to activate and rapidly proliferate. Myeloablative preconditioning can cause changes, such as intestinal epithelial damage and inflammation in recipients, significantly altering the hematopoietic environment compared with that during homeostasis. Therefore, post-transplant hematopoiesis differs from hematopoiesis under homeostasis [10, 11]. Studying post-transplant hematopoiesis in conjunction with reduced dietary intake will enable better understanding the biological features of HSCs under proliferation pressure and their responses to a metabolically regulated environment.

In clinical practice, myeloablative preconditioning often causes nausea, vomiting, appetite loss, severe oral ulcers, gastrointestinal damage and gastroenteritis in patients, often leading to greatly reduced caloric uptake in these patients [12, 13]. However, the impact of these common complications on HSCs and post-transplant hematopoiesis and their potential clinical significance remain unclear.

In this study, C57BL/6J mice were irradiated with a lethal X-ray dose followed by bone marrow transplantation. After transplantation, we exposed the recipient mice to either a 30% DR or an ad libitum (AL) diet to study the role of reduced dietary intake on post-transplant hematopoiesis. Post-transplant DR markedly inhibited hematopoietic reconstitution, with both lymphoid and myeloid hematopoiesis significantly delayed in the recipients. However, when the reconstituted bone marrow cells (BMCs) were serially transplanted to secondary or tertiary recipient mice fed the AL diet, the BMCs derived from the mice primarily subjected to post-transplant DR showed a significantly enhanced hematopoietic reconstitution capacity. Furthermore, we transplanted purified HSCs and found that post-transplant DR clearly inhibited HSC expansion, which may explain why hematopoietic reconstitution was delayed in the recipients while post-transplant DR enhanced the hematopoietic reconstitution capacity of the HSCs.

## Materials and Methods

### Mice and Diets

Three-month-old C57BL/6J mice were obtained from Hunan SJA Laboratory Animal Co., Ltd. and maintained in the animal facilities of Nanchang Royo Biotech under pathogen-free conditions on a 12-h light/12-h dark cycle at 23–25 °C. The mice were housed individually and received a regimen of either an AL diet (fed an unlimited amount of food) or a DR diet (fed daily with an amount of food corresponding to 70% of the amount of food consumed by body weight- and gender-matched mice in the AL group) after bone marrow transplantation. The food amount provided remained constant over the entire DR period. The Animal Experimental Ethical Inspection of Nanchang Royo Biotech Co., Ltd. (RYEI20170513-1) approved all mouse experiments.

### Transplantation

For the primary bone marrow transplantation, 1 million BMCs from Ly5.1 donor mice were injected via tail vein into lethally irradiated (X-ray, 9 Gy) Ly5.2 recipient mice. Bone marrow and peripheral blood analyses were performed at 1 and 4 months post-transplantation. For the secondary transplantation, 8 million BMCs from the post-transplant DR or AL mice along with 2 million BMCs from Ly5.2 mice (competition cells) were injected via tail vein into lethally irradiated Ly5.2 recipient mice. For the tertiary transplantation, 10 million BMCs from the secondary recipients were injected via tail vein into lethally irradiated Ly5.2 recipient mice. Bone marrow and peripheral blood analyses of the secondary and tertiary transplantation were performed 4 months after transplantation.

For HSC transplantation, 2000 FACS-purified HSCs from Ly5.1 donor mice were injected via tail vein into lethally irradiated Ly5.2 recipient mice. Bone marrow and peripheral blood analyses were performed 3 weeks after transplantation.

### Flow Cytometry

BMCs were obtained by crushing the hind limbs and pelvises of the donor mice in sterile phosphate-buffered saline and filtered with a 40-μm cell strainer. BMCs were resuspended in red cell lysis buffer and incubated at room temperature for 5 min to lyse the red blood cells. Afterward, the cells were washed, counted, and incubated with flow cytometric antibodies. HSCs were detected using a lineage cocktail (biotinylated anti-TER-119, -Gr-1, -B220, -CD11b, -CD3, -CD4 and -CD8), streptavidin-APC-Cy7, CD45.1-PE, c-Kit-APC, Sca-1-PE-Cy7, CD150-PerCP-Cy5.5, and CD34-FITC (BD). Common myeloid progenitors (CMPs), megakaryocyte/erythrocyte progenitors (MEPs) and granulocyte/macrophage (GMPs) were detected using a lineage cocktail (biotinylated anti-TER-119, -Gr-1, -B220, -CD11b, -CD3, -CD4 and -CD8), streptavidin-APC-Cy7, CD45.1-PE, c-Kit-APC, Sca-1-PE-Cy7, CD34-FITC (BD), and CD16/32-BV421. Common lymphoid progenitor cells (CLPs) were detected using a lineage cocktail (biotinylated anti-TER-119, -Gr-1, -B220, -CD11b, -CD3, -CD4 and -CD8), streptavidin-APC-Cy7, CD45.1-FITC, c-Kit-APC, Sca-1-PE-Cy7, CD127-PerCP-Cy5.5, and CD135-PE. Pro-B cells were detected using a lineage cocktail (biotinylated anti-TER-119, -Gr-1, -CD11b, and -CD3), streptavidin-APC-Cy7, CD45.1-PE, CD19-PerCP-Cy5.5, B200-PE-Cy7, AA4.1-APC, and CD24-FITC. Differentiated BMCs were detected using CD45.1-FITC, B200-PE-Cy7 and CD11b-APC-Cy7. Thymocytes were detected using CD45.1-FITC, CD4-APC and CD8a-PerCP-Cy5.5. Thymocytes and peripheral blood cells were detected using CD45.1-FITC, B200-PE-Cy7, CD11b-APC-Cy7, CD4-APC, and CD8a-PerCP-Cy5.5.

For chimerism analyses, HSCs were detected using a lineage cocktail (biotinylated anti-TER-119, -Gr-1, -B220, -CD11b, -CD3, -CD4 and -CD8), streptavidin-APC-Cy7, CD45.1-PE, c-Kit-APC, Sca-1-PE-Cy7, CD150-PerCP-Cy5.5, and CD34-FITC (BD). Differentiated BMCs were detected using CD45.1-PE, CD45.2-FITC, B200-PE-Cy7, and CD11b-APC-Cy7. Peripheral blood cells were detected using CD45.1-PE, CD45.2-FITC, B200-PE-Cy7, CD11b-APC-Cy7, CD4-APC, and CD8a-PerCP-Cy5.5. After staining, cells were analyzed on a flow cytometer (FACS Canto II; BD). All antibodies were obtained from BioLegend unless otherwise noted.

### HSC Sorting

BMCs were incubated with c-Kit-APC, and c-Kit + cells were enriched using anti-APC magnetic beads and LS columns (Miltenyi Biotec). The positively selected cells were then stained for HSC markers using a lineage cocktail as described in the previous section and CD34-FITC (BD), CD150-PerCP-Cy5.5, Kit-APC, and Sca-1-PE and streptavidin-APC-Cy7. After staining, cells were sorted on a cell sorter (FACS Aria III; BD). All antibodies were obtained from BioLegend unless otherwise noted.

### Histology

Femurs from the post-transplant DR and AL mice were collected and fixed in 4% paraformaldehyde for 24 h. Fixed samples were processed and embedded in paraffin using standard protocols, and 5-μm paraffin sections were prepared. Bone section histology was assessed by hematoxylin and eosin (H&E) staining. Representative areas were photographed using an OLYMPUSIX73 microscope (Japan).

### Peripheral Blood Cell Counting

Peripheral blood cells were collected from the orbital venous plexus, placed in 5 μL 0.5 M EDTA, and counted on an automated hematology analyzer (Sysmex, XS-500i) according to the manufacturer’s instructions.

### Fecal Sample Collection

Fresh fecal samples were directly collected from each mouse by positioning the microtube in the proximity of the anus of the mouse. Excreted fecal pellets were collected in microtubes on ice and stored at -80 °C within 1 h until DNA isolation for 16 S rRNA gene sequencing.

### Fecal DNA Isolation

Fecal samples were weighed and total DNA was extracted with the DP328 Fecal Genome Extraction Kit (Tiangen Biotech) according to the manufacturer’s instructions. DNA concentration and purity were measured with Nanodrop 2000.

### 16 S rRNA Gene Sequencing

Fecal-sample DNA was extracted using DNA extraction kit. The concentration and purity were measured using the NanoDrop One (Thermo Fisher Scientific, MA, USA). 16 S rRNA gene regions V3-V4 were amplified using universal primers [14] (mice: 338 F 5ʹ-ACTCCTACGGGAGGCAGCA-3ʹ and 806R 5ʹ-GGACTACHVGGGTWTCTAAT-3ʹ) with 12 bp barcode, Primers were synthesized by Invitrogen (Invitrogen, Carlsbad, CA, USA). PCR reactions, containing 25 μl 2x Premix Taq (Takara Biotechnology, Dalian Co. Ltd., China), 1 μl each primer(10 μM) and 3 μl DNA (20 ng/μl) template in a volume of 50 μl, were amplified by thermocycling: 5 min at 94 °C for initialization; 30 cycles of 30 s denaturation at 94 °C, 30 s annealing at 52 °C, and 30 s extension at 72 °C; followed by 10 min final elongation at 72 °C. The PCR instrument was BioRad S1000 (Bio-Rad Laboratory, CA, USA).

The length and concentration of the PCR products were detected by 1% agarose gel electrophoresis. PCR products were mixed in equimolar ratios according to the GeneTools Analysis Software (Version4.03.05.0, SynGene). Then, the PCR mixture was purified with EZNA Gel Extraction Kit (Omega, USA). Sequencing libraries were generated using NEBNext^®^ Ultra™ DNA Library Prep Kit for Illumina^®^ (New England Biolabs, USA) following the manufacturer’s recommendations and index codes were added. The library quality was assessed on the Qubit 2.0 Fluorometer (Thermo Scientific). Finally, the library was sequenced on an Illumina Nova6000 platform and 250 bp paired-end reads were generated.

### Sequencing Data Processing

#### Paired-end Raw Reads Quality Control

Fastp (version 0.14.1, https://github.com/OpenGene/fastp) was used to control the quality of the Raw Data by sliding window(-W 4 -M 20). The primers were removed by using cutadapt software (https://github.com/marcelm/cutadapt/) according to the primer information at the beginning and end of the sequence to obtain the paired-end clean reads.

#### Paired-end Clean Reads Assembly

Paired-end clean reads were merged using usearch -fastq_mergepairs (V10,http://www.drive5.com/usearch/)according to the relationship of the overlap between the paired-end reads, when at least 16 bp overlap the read generated from the opposite end of the same DNA fragment, the maximum mismatch allowed in overlap region was 5 bp, and the spliced sequences were called Raw Tags.

#### Raw Tags Quality Control

Use fastp to remove low-quality sequences, calculate the average quality values for the bases in the sliding window, and then remove the non-conforming sliding windows. The -W parameter specifies the size of the sliding window, which is 4 by default, and the -M parameter is used to specify the required average quality value, which is 20 by default, which is Q20. Fastp (version 0.14.1, https://github.com/OpenGene/fastp) was used to control the quality of the raw Data by sliding window(-W 4 -M 20) to obtain the paired-end clean tags.

### OTU Cluster and Species Annotation

OTU (operational taxonomic units) is one of the most common terms in microbiology. Sequences analysis was performed by usearch software (V10, http://www.drive5.com/usearch/). Sequences with ≥ 97% similarity were assigned to the same OTU. An OTU is thought to possibly represent a species. The most frequently occurring sequence was extracted as a representative sequence for each OTU and was screened for further annotation.

### Statistical Analysis

GraphPad Prism 9.0 software was used for statistical analysis. Unpaired two-tailed Student’s t-tests were used for two-group datasets to calculate *p*-values. All results are displayed as means ± standard deviation (SD). *p < 0.05; **p < 0.01; ***p < 0.001; ****p < 0.0001; ns, not significant.

## Results

### Post-transplant DR Significantly Delayed Hematopoietic Reconstitution

**Fig. 1:**
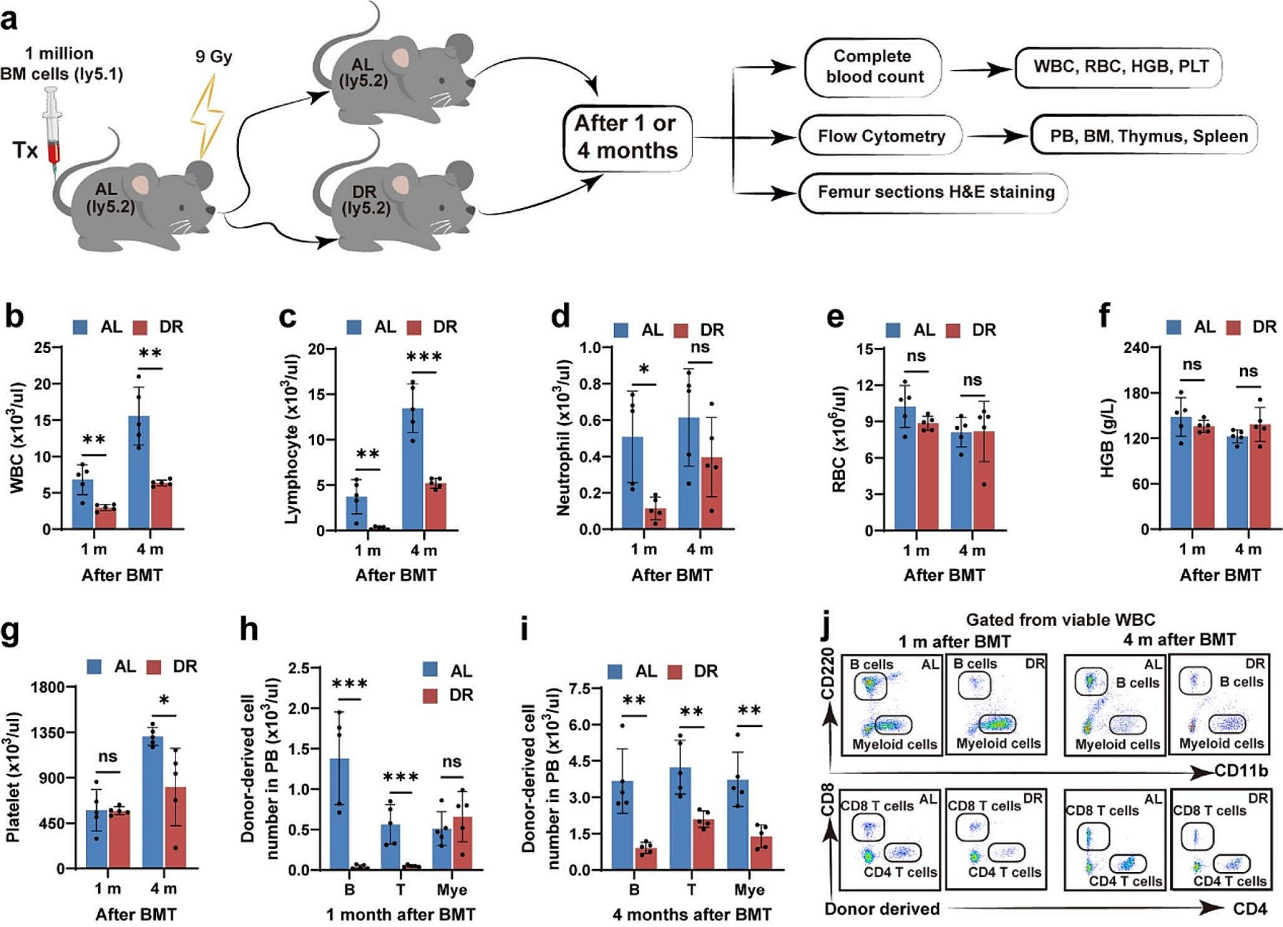
Post-transplant DR significantly delayed peripheral blood regeneration Three-month-old mice were fed a DR or AL diet after transplantation. Peripheral blood analysis was performed at 1 and 4 months after transplantation. (**a**) Experimental procedure of bone marrow transplantation. (**b–g** ) Peripheral blood analysis. (**h–j**) Flow cytometry analysis of donor-derived WBC subsets and representative photographs. *n* = 5 mice per group randomly picked from two independent experiments. Results are displayed as means ± SD. *p < 0.05; **p < 0.01; ***p < 0.001; ns: not significant by unpaired, two-tailed, Student’s t-tests. AL, ad libitum; DR, dietary restriction; WBC, white blood cell; RBC, red blood cell; HGB, hemoglobin; PLT, platelet; PB, peripheral blood; BM, bone marrow; BMT, bone marrow transplantation; SD, standard deviation

To investigate the effect of reduced food intake on post-transplant hematopoiesis, we treated recipient mice with 30% DR directly after bone marrow transplantation (Fig. 1a). Peripheral blood cell counting at 1 and 4 months after transplantation showed that the numbers of white blood cells (WBCs) and lymphocytes remained markedly low in the DR-recipient mice compared with those of recipient mice fed the AL diet, although the red blood cells and platelets were unaltered (Fig. 1b–g). Flow cytometry analysis further showed that reconstitution of donor-derived peripheral B and T cells was severely inhibited in DR recipients both at 1 and 4 months post-transplantation, and the regeneration of donor-derived myeloid cells was also significantly delayed in DR recipients 4 months after transplantation (Fig. 1h–j, Fig. S1a-d for representative gating strategy).

**Fig. 2:**
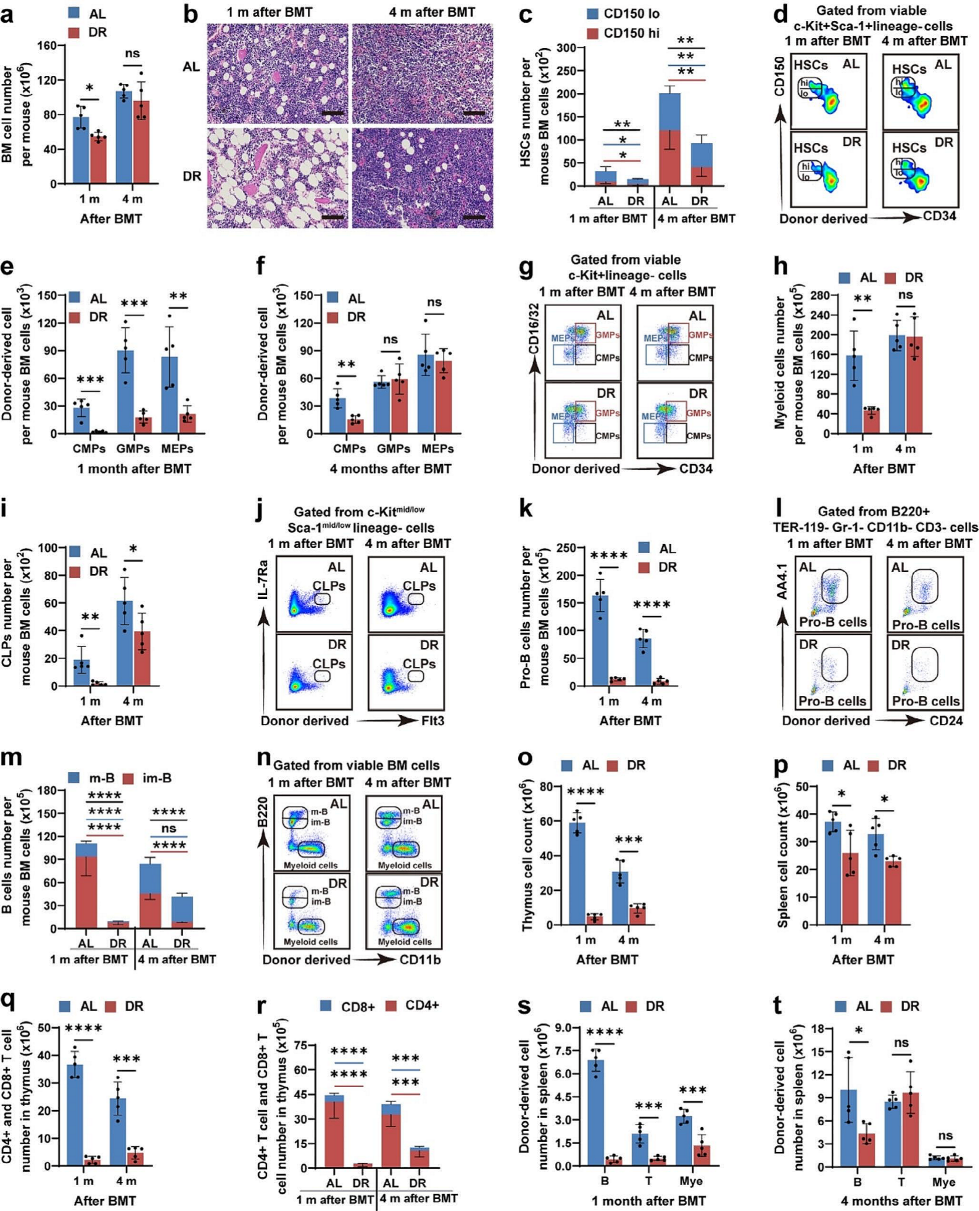
Post-transplant DR significantly delayed hematopoietic reconstitution in bone marrow Three-month-old mice were fed a DR or AL diet after transplantation. Analysis on bone marrow cells was performed at 1 and 4 months after transplantation. (**a, b**) Total bone marrow cellularity and femur sections with H&E staining. Scale bar: 100 μm. (**c–n**) Flow cytometry analysis of donor-derived HSCs, CMPs, GMPs, MEPs, CPLs, Pro-B cells, B cells and myeloid cells and representative photographs. (**o–t**) Thymocyte and splenocyte counts and flow cytometry analysis of thymocyte and splenocyte subsets. *n* = 5 mice per group randomly picked from two independent experiments. Results are displayed as means ± SD. *p < 0.05; **p < 0.01; ***p < 0.001; ****p < 0.0001; ns: not significant by unpaired, two-tailed, Student’s t-tests

Recipient mice were euthanized for further analysis at 1 and 4 months after transplantation. Bone marrow cellularity was reduced in DR recipients 1 month after transplantation (54.72 ± 4.88 million cells per mouse in DR recipients versus 77.00 ± 12.57 million cells per mouse in AL recipients); however, they became similar in AL- and DR-recipient mice 4 months post-transplantation (Fig. 2a). H&E staining of femur sections also showed reduced cellularity and increased number of lipids in the bone marrow of DR mice 1 month after transplantation, while the cellularity become similar between both groups with lipids remaining increased in DR recipients 4 months after transplantation (Fig. 2b). Interestingly, the frequencies of total HSCs (CD150^+^CD34^−^ c-Kit^+^Sca-1^+^lineage^−^ cells), myeloid-biased HSCs (CD150^high^ HSCs) and lymphoid-biased HSCs (CD150^low^ HSCs) were significantly lower in DR mice at both 1 and 4 months post-transplantation (Fig. 2c, d, Fig. S2a-d). Flow cytometry analysis of BMCs showed that frequencies of CMPs (CD16/32^−^CD34^+^c-Kit^+^Sca-1^−^lineage^−^ cells), MEPs (CD16/32^−^CD34^−^c-Kit^+^Sca-1^−^lineage^−^ cells) and GMPs (CD16/32^+^CD34^+^c-Kit^+^Sca-1^−^lineage^−^ cells) were significantly lower in DR recipients at 1 month after transplantation, and the frequency of CMPs remained significantly lower at 4 months after transplantation (Fig. 2e–g, Fig. S2e-h). Consistently, the frequencies of downstream myeloid cells (CD11b^+^ BMCs) were also reduced in DR recipients 1 month after transplantation, but became similar in the AL- and DR-fed mice at 4 months after transplantation, indicating significantly delayed myeloid lineage reconstitution (Fig. 2h). The lymphoid lineages, including CLPs (IL-7Rα^+^Flt3^+^c-Kit^mid/low^Sca-1^mid/low^lineage^−^ cells), pro-B cells (B220^+^CD24^+^AA4.1^+^TER-119^−^Gr-1^−^CD11b^−^CD3^−^ cells), and B cells in bone marrow (B220^low^ immature B cells) also showed greatly reduced frequencies in DR recipients at 1 and 4 months after transplantation (Fig. 2i–n, Fig. S2i-t). Consistent with the significant inhibition of lymphoid cells in the peripheral blood and bone marrow, cellularity of lymphoid organs, including the thymus and spleen, remained significantly lower in DR recipients at 1 and 4 months after transplantation (Fig. 2o, p). Flow cytometry analysis further showed that frequencies of donor-derived cells were significantly lower in these organs in DR recipients (Fig. 2q–t). *These results indicated that post-transplant DR significantly inhibited both myeloid and lymphoid hematopoietic reconstitution.*

### Post-transplant DR Improved the Reconstitution Potential of BMCs in Serial Transplantations

**Fig. 3:**
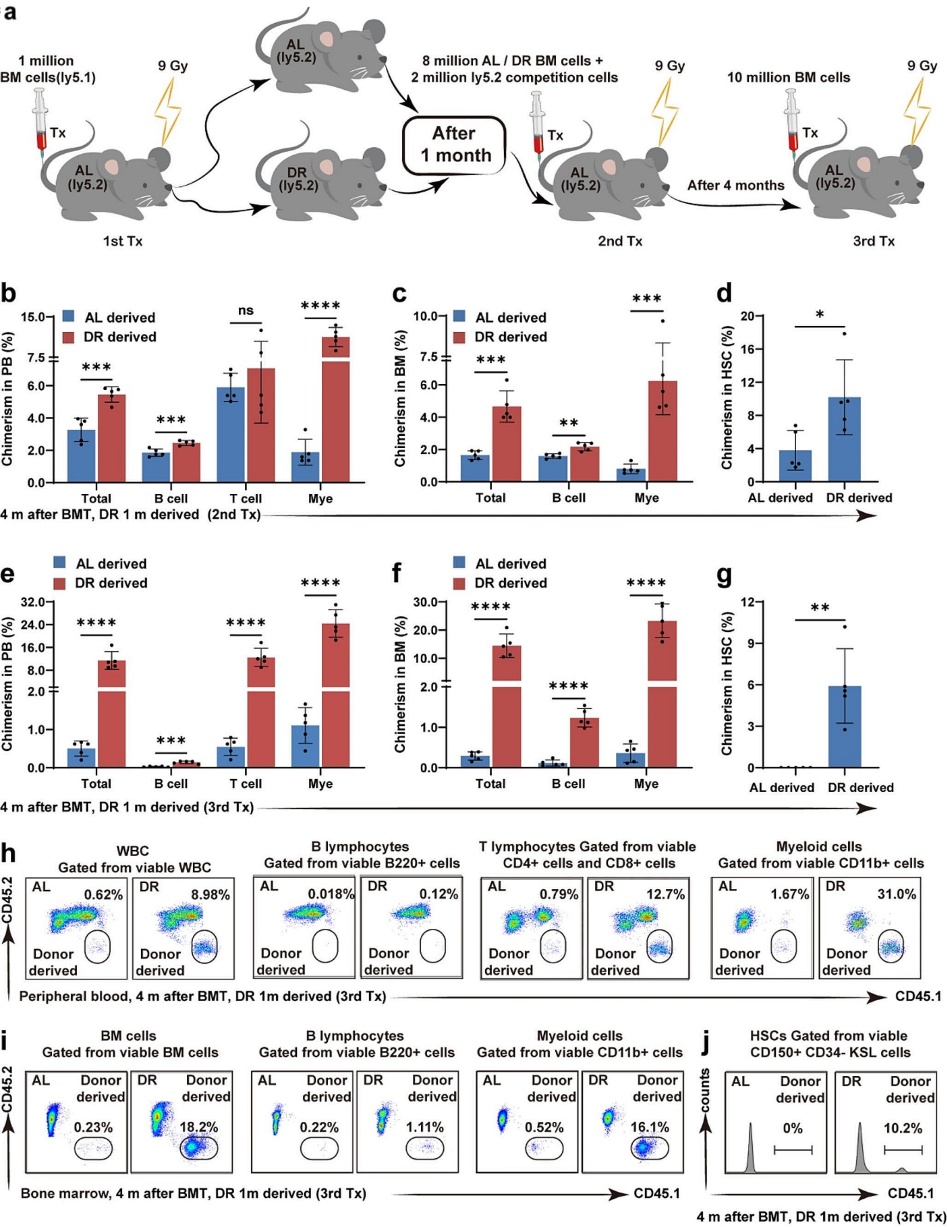
Post-transplant DR for 1 month improved reconstitution potential of BMCs in serial transplantations In the secondary transplantation, BMCs from post-transplant DR or AL recipients at 1 month were injected via tail vein into lethally irradiated (X-ray, 9 Gy) Ly5.2 recipient mice. In the tertiary transplantation, BMCs from the secondary recipients were injected 4 months after transplantation via tail vein into lethally irradiated Ly5.2 recipient mice. Bone marrow and peripheral blood analyses were performed 4 months after secondary and tertiary transplantation. (**a**) Experimental procedure of bone marrow transplantation. (**b–d, h**) Chimerism of donor-derived cells in the peripheral blood and bone marrow 4 months after secondary transplantation and representative photographs. (**e-g, i, j**) Chimerism of donor-derived cells in the peripheral blood and bone marrow 4 months after tertiary transplantation and representative photographs. *n* = 5 mice per group randomly picked from two independent experiments. *p < 0.05; **p < 0.01; ***p < 0.001; ****p < 0.0001; ns: not significant by unpaired, two-tailed, Student’s t-tests

Post-transplant energy restriction by DR severely impaired hematopoietic regeneration in the recipient mice. To investigate the effect of post-transplant DR on the reconstitution potential of regenerated BMCs in recipient mice, mice were fed an AL or DR diet after transplantation, and the regenerated BMCs were harvested 1 month after transplantation and serially transplanted along with competitor cells into secondary and tertiary recipient mice fed AL after transplantation (Fig. 3a). In the secondary transplantation, DR-recipient donor-derived bone marrow showed significantly higher outputs of peripheral WBCs, *B cells*, and myeloid cells at 4 months after the secondary transplantation in both the peripheral blood and bone marrow (Fig. 3b, c). Interestingly, DR-derived HSCs also showed significantly higher chimerism (Fig. 3d). In the tertiary transplantation, DR-recipient donor-derived bone marrow was superior in the post-transplant hematopoietic reconstitution with greatly enhanced outputs of peripheral WBCs, lymphocytes, and myeloid cells in the peripheral blood and bone marrow (Fig. 3e, f, h, i, Fig. S3a-d). DR-derived HSCs also exhibited significantly higher chimerism than that of AL-derived HSCs, which was nearly undetectable after tertiary transplantation (Fig. 3g, j, Fig. S3e-f). Because the HSC frequency in the reconstituted bone marrow after primary transplantation was significantly lower in DR recipients than in AL recipients (Fig. 2c), the results indicate that compared with AL feeding, post-transplant DR for 1 month enhanced the regeneration potential of the reconstituted bone marrow. Thus, we inferred that short-term post-transplant DR (for 1 month) strengthened the regeneration potential of the reconstituted HSCs.

**Fig. 4:**
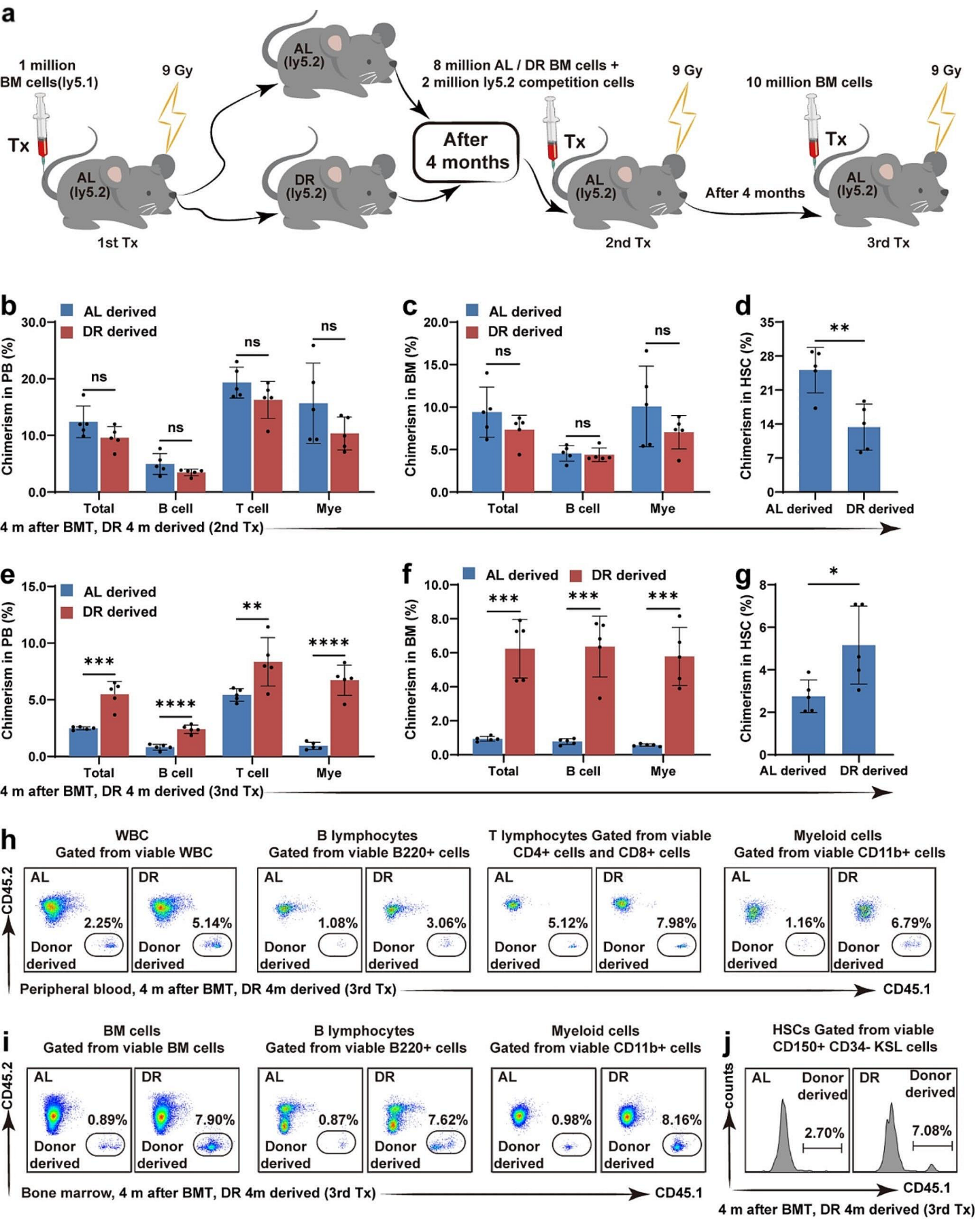
Post-transplant DR for 4 months improved reconstitution potential of BMCs in serial transplantations In the secondary transplantation, BMCs from post-transplant DR or AL recipients after 4 months were injected via tail vein into lethally irradiated Ly5.2 recipient mice. In the tertiary transplantation, BMCs from the secondary recipients were injected 4 months after transplantation via tail vein into lethally irradiated Ly5.2 recipient mice. Bone marrow and peripheral blood analyses were performed 4 months after the secondary and tertiary transplantation. (**a**) Experimental procedure of bone marrow transplantation. (**b-d, h**) Chimerism of donor-derived cells in the peripheral blood and bone marrow 4 months after secondary transplantation and representative photographs. (**e–g, i, j**) Chimerism of donor-derived cells in the peripheral blood and bone marrow 4 months after tertiary transplantation and representative photographs. *n* = 5 mice per group randomly picked from two independent experiments. *p < 0.05; **p < 0.01; ***p < 0.001; ****p < 0.0001; ns: not significant by unpaired, two-tailed, Student’s t-tests

We previously showed that longer term DR had a stronger effect on improving the reconstitution potential of HSCs in aging mice under homeostasis conditions [9]. To investigate the effect of a longer post-transplant DR on the reconstitution potential of regenerated BMCs in recipient mice, mice were fed either an AL or DR diet after transplantation. The regenerated BMCs were then harvested 4 months after transplantation and serially transplanted along with competitor cells into secondary and tertiary recipient mice, which were fed AL after transplantation (Fig. 4a). Surprisingly, the longer post-transplantation DR (4 months) did not improve the reconstitution of WBCs, lymphocytes or myeloid cells in the peripheral blood or bone marrow in the secondary transplantation (Fig. 4b, c). DR-recipient donor-derived BMCs even showed significantly reduced chimerism in HSCs compared with that of the AL-recipient donor-derived BMCs (Fig. 4d). In the tertiary transplantation, DR-recipient donor-derived bone marrow showed higher outputs of WBCs, lymphocytes, and myeloid cells in the peripheral blood and bone marrow (Fig. 4e, f, h, i, Fig. S4a-d). The HSC chimerism from the DR-derived donors was also significantly increased compared with that from AL-derived donors (Fig. 4g, j, Fig. S4e-f). These results indicated that short-term DR (1 month) after transplantation significantly improved the reconstitution of WBCs, lymphocytes, and myeloid cells in the peripheral blood or bone marrow, as well as HSCs, in the secondary and tertiary transplantations. However, long-term DR (4 months) after transplantation differed from long-term DR under steady-state conditions, showing no significant improvement in the secondary transplantation but significantly improving reconstitution in the tertiary transplantation.

### Post-transplant DR Significantly Inhibited HSC Expansion and Delayed Hematopoietic Reconstitution

**Fig. 5:**
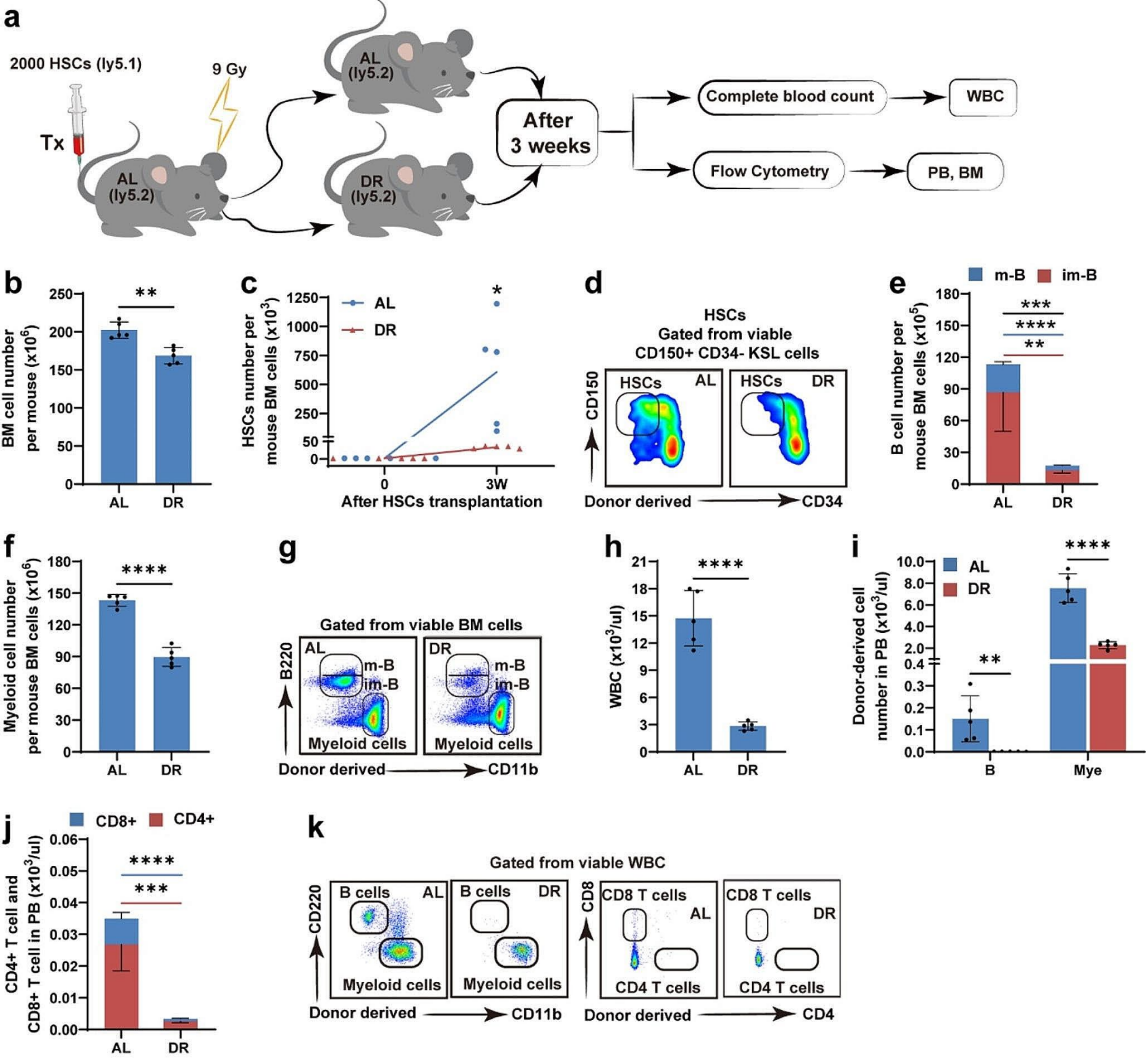
Post-transplant DR significantly inhibited expansion of HSCs and delayed hematopoietic reconstitution 2000 FACS-purified HSCs from Ly5.1 donor mice were injected via tail vein into lethally irradiated Ly5.2 recipient mice. Bone marrow and peripheral blood analyses were performed 3 weeks after transplantation. (**a**) Experimental procedure of HSC transplantation. (**b**) Total bone marrow cellularity. (**c–g**) Flow cytometry analysis of donor-derived HSCs, B cells and myeloid cells and representative photographs. (**h**) Peripheral blood analysis. (**i–k**) Flow cytometry analysis of donor-derived WBC subsets and representative photographs. *n* = 5 mice per group randomly picked from two independent experiments. *p < 0.05; **p < 0.01; ***p < 0.001; ****p < 0.0001 by unpaired, two-tailed, Student’s t-tests

To further investigate the underlying mechanism by which post-transplant DR protects the regeneration activities of HSCs while delaying post-transplant hematopoietic reconstitution, pure HSCs were sorted by FACS and transplanted at 2000 cells/mouse into recipient mice fed AL or DR after transplantation. Hematopoietic reconstitution was analyzed 3 weeks after transplantation (Fig. 5a). Bone marrow cellularity was mildly but significantly reduced in the DR-recipient mice (Fig. 5b). The number of donor-derived HSCs was 608,300 per mouse, representing a 304-fold expansion compared with that of the 2000 HSCs initially transplanted in the AL recipients. Conversely, the DR recipients’ HSC count was only 33,900 per mouse, indicating a 17-fold expansion in these mice at this time point, suggesting that post-transplant DR strongly inhibited HSC expansion (Fig. 5c, d, Fig. S5a-b). Consistently, the numbers of B cells and myeloid cells in the bone marrow were significantly lower in DR recipients (Fig. 5e–g, Fig. S5c-d). The peripheral blood of DR recipients contained markedly fewer WBCs, donor-derived B cells, myeloid cells, CD4 + T cells, and *CD8 + T cells* (Fig. 5h–k, Fig. S5e-f). These results indicated that post-transplant DR significantly inhibited HSC expansion and delayed hematopoietic reconstitution.

### Post-transplant DR Significantly Altered the Gut Microbiotas of the Recipient Mice

**Fig. 6:**
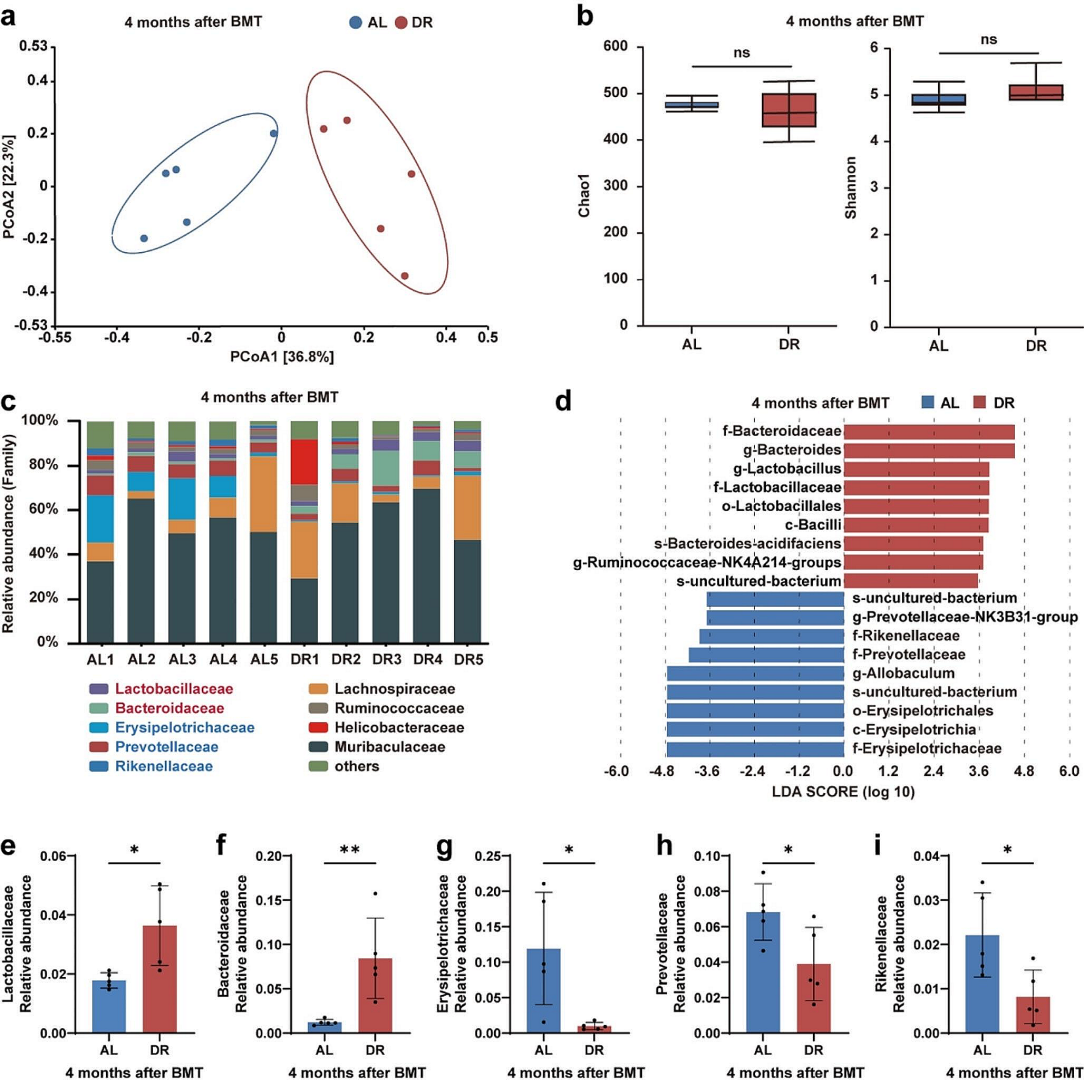
Post-transplant DR significantly altered the gut microbiotas of the recipient mice Fecal samples were randomly collected from post-transplant DR or AL mice at 4 months, and gut microbiotas were analyzed by 16 S rRNA gene sequencing. (**a**) Principal coordinate analysis based on Bray-Curtis distance showed a significant shift of the overall gut microbiota structure by post-transplant DR. (**b**) Measures of alpha diversity: Chao1 and shannon’s diversity index. (**c**) Relative abundances of the *gut microbiota* of indicated groups at the *family level* as per 16 S rRNA gene sequencing. (**d**) LDA scores in the fecal microbiomes of indicated groups. LDA scores > 3.5 and the *top 18 bacteria* are shown. (**e–i**) Relative abundances of Lactobacillaceae, Bacteroidaceae, Erysipelotrichaceae, Prevotellaceae, and Rikenellaceae as per 16 S rRNA gene sequencing. *n* = 5 mice per group randomly picked from two independent experiments. *p < 0.05; **p < 0.01; ns: not significant by unpaired, two-tailed, Student’s t-tests

We previously reported that increased Bacteroidaceae mediated lymphopoiesis inhibition in DR mice under homeostasis conditions [15]. We also found that Lactobacillales conferred a protective effect on chemotherapy-induced intestinal toxicity by downregulating inflammatory responses [16–19]. Furthermore, studies have shown that the gut microbiota plays important roles in hematopoiesis after bone marrow transplantation [2]. To investigate the effect of post-transplantation DR on the gut microbiota, the recipient mice were fed AL or a DR diet for 4 months after bone marrow transplantation, and their feces were collected for 16 S rRNA gene deep-sequencing (Illumina; 250-bp paired-end). Principal coordinate analysis based on Bray-Curtis distance showed that DR significantly changed the overall structure of the gut microbiota (Fig. 6a). However, Chao1 and Shannon indices were not significantly different between DR and AL mice, indicating that microbial diversity (alpha diversity) was not altered by post-transplant DR (Fig. 6b). These results indicated that DR significantly altered the structure of the gut microbiota without affecting its diversity, including species richness and evenness. Furthermore, analysis of the gut microbiota composition at the family level showed higher percentages of pathogenic bacteria (e.g., Betaproteobacterales and Enterobacteriales) in DR-recipient mice (Fig. 6c). We then used linear discriminant analysis (LDA) scores > 3.5 and looked at the *top 18 bacteria* in each direction. Bacteroidaceae, *Bacteroides, Lactobacillus*, Lactobacillaceae, and Lactobacillales were among the top 5 enriched taxa in DR mice compared with the AL mice (Fig. 6d). Statistical analysis further indicated significantly increased relative abundances of Bacteroidaceae and Lactobacillales in the gut microbiotas of the DR-recipient mice (Fig. 6e, f). Conversely, DR decreased the relative abundances of Erysipelotrichaceae, Prevotellaceae and Rikenellaceae in the gut microbiotas of the DR-recipient mice (Fig. 6g–i). The results indicate that post-transplant DR increased the relative abundance of anti-inflammatory bacteria in the gut of mice, including Bacteroidaceae and Lactobacillaceae, and decreased the relative abundance of pro-inflammatory bacteria, including Erysipelotrichaceae, Prevotellaceae, and Rikenellaceae.

## Discussion

In this study, we, for the first time, systematically investigated the role of post-transplant DR on hematopoietic reconstitution and HSC function after HCT. Post-transplant DR significantly inhibited hematopoietic reconstitution, including lymphopoiesis and myelopoiesis. However, post-transplant DR significantly protected the regeneration capacity of HSCs. Furthermore, the transplantation assay with pure HSCs showed that the HSC pool greatly expanded after transplantation under AL conditions, reflecting the physiological behavior of HSCs after transplantation. This may explain the decreased HSC regeneration capacity in serial transplantations. However, DR significantly inhibited HSC expansion after transplantation, which partially explained its protective effect on HSC function. Previous studies have reported that the gut microbiota plays an important role in post-transplant hematopoiesis [2, 20]. We and other researchers have conducted extensive studies on gut microbiota, DR, and HSCs. Our early studies reported that DR under steady-state conditions can delay and rejuvenate HSC aging and improve regenerative functions of HSCs. Subsequently, we found that short-term DR before MTX or 5-FU chemotherapy can significantly alter the composition of the gut microbiota, such as promoting the upregulation of beneficial bacteria like *Lactobacillus* and inhibiting opportunistic pathogens like Proteus mirabilis and Enterococcus, thereby protecting intestinal epithelial cells and blocking bacterial translocation and corresponding lethal infections [19, 21]. We also reported that DR under steady-state conditions can increase intestinal*Bacteroides* and inhibiting lymphoid hematopoiesis by promoting butyrate utilization [15]. DR in aging mice can significantly alter their gut microbiota composition, making it more similar to that of young mice. Numerous previous studies have also revealed the important role of gut microbiota in hematopoiesis. Zhang et al. reported that gut microbiota is crucial for hematopoietic recovery after 5-FU chemotherapy or radiotherapy, as depletion of the gut microbiota can significantly delay hematopoietic recovery [22]. Huang et al. found that gut microbiota is crucial for HSC aging, with the gut microbiota of aging mice promoting HSC aging, while the gut microbiota of young mice can reverse HSC aging in elderly mice [23]. In allogeneic hematopoietic stem cell transplantation (allo-HSCT), gut microbiota also plays a key role in the development of graft-versus-host disease [24–26]. Furthermore, gut microbiota plays a critical role in food breakdown and energy absorption, which are vital for hematopoietic recovery after transplantation. However, these studies have not explored the effects of DR on gut microbiota, HSCs, and hematopoietic recovery after HSCT. The current study provides preliminary and worthy exploration in this area, suggesting that DR after HSCT can alter the gut microbiota composition and reduce pro-inflammatory bacteria. Although we have not yet conducted in-depth studies on the causal relationship between these changes in gut microbiota and HSC function and hematopoietic recovery after transplantation, we provide interesting preliminary findings that can be further explored in future research.

We found that post-transplant DR significantly changed the gut microbiota composition. We previously reported that increased intestinal *Bacteroides* is a major factor mediating lymphopoiesis inhibition by DR. Our results showed that post-transplant DR significantly increased intestinal *Bacteroides*, which may help suppress hematopoietic reconstitution via DR after transplantation. Erysipelotrichaceae, Prevotellaceae and Rikenellaceae are reported to help promote inflammation. We and others have reported that DR can significantly suppress inflammatory responses [19, 27, 28]. Specifically, short-term DR before methotrexate treatment increased Lactobacillales in the gut, which ameliorated intestinal inflammation and improved survival [19]. In the current study, we showed that post-transplant DR significantly regulated inflammatory-related bacterial taxa, including increased Lactobacillales and decreased Erysipelotrichaceae, Prevotellaceae and Rikenellaceae, which may inhibit inflammatory responses after transplantation, thus protecting HSCs. However, patients undergoing HCT are exposed to various factors that can disrupt the gut microbiota before transplantation, during the peri-transplant period, and after transplantation, including chemotherapy, antibiotics, and dietary changes. These disruptions result in changes in gut microbiota profile characterized by a loss of microbial diversity, dysbiosis, and the expansion of opportunistic pathogens such as Enterococcus, Streptococcus, or Proteus compared to healthy individuals [24]. In contrast, in the bone marrow transplantation mouse model, changes in the gut microbiota are only slight. A major reason of the difference is considered to be patients frequently use antibiotics, whereas antibiotics are not commonly used in mice [24]. More clinical studies are needed to systematically analyze HSCs and their downstream cells in patients undergoing DR and to explore the underlying mechanisms. However, there are practical difficulties in administering DR in patients and collecting samples, making comprehensive analysis unlikely to be achievable in the short term. The significance of this study lies in its systematic investigation of the effects of post-transplant DR on the hematopoietic system, particularly on HSCs, from short-term to long-term perspectives at both phenotypic and cellular levels. Moreover, due to differences between humans and mice in metabolism, immune response, and gut microbiota, it is currently challenging to directly apply these findings to clinical practice, necessitating more in-depth translational research.

We previously reported that long-term DR (6–9 months) under homeostasis conditions significantly inhibited lymphoid hematopoiesis and promoted myeloid hematopoiesis. However, when transplanted into AL-recipient mice, DR-donor-derived HSCs showed a significantly enhanced reconstitution capacity, especially in the lymphoid lineage, indicating rescue of the HSC aging phenotype, including myeloid skewing and functional decline. In the current study, we investigated the role of DR in post-transplant hematopoiesis. Unlike hematopoiesis under homeostasis conditions, in HCT, the original hematopoietic system in recipients must be cleared by myeloablation treatments. Therefore, the donor-derived hematopoietic cells are challenged with rebuilding the hematopoietic system when transplanted into recipients. We found that post-transplant DR significantly inhibited lymphoid hematopoiesis and inhibited myeloid hematopoiesis in recipients. Moreover, the frequencies of both myeloid-biased (CD150^hi^) and lymphoid-biased (CD150^lo^) HSCs were significantly reduced, as were the frequencies of myeloid progenitor cells (CMPs, GMPs, and MEPs) and lymphoid progenitor cells (CLPs and pro-Bs). The delayed reconstitution of the entire hematopoietic system by DR under post-transplant conditions differed from that of enhanced myeloid hematopoiesis by DR under homeostasis conditions. That DR significantly and comprehensively delays hematopoietic reconstruction has important significance for clinical practice. Patients who receive HCT often suffer from anorexia, aphthous ulcers and gastrointestinal damage due to the toxicity induced by the conditioning regimen, which reduces their appetite and weakens their absorption and digestive functions. Our results indicate that post-transplant diet and calorie management greatly affect hematopoietic reconstruction, which may play crucial roles in transplantation-related complications, such as infections. Therefore, our findings may have important implications for clinical work and should draw attention to post-transplant calorie management.

Post-transplant DR significantly protected and improved HSC functions, although it significantly delayed hematopoietic reconstruction. Sequentially transplanting BMCs from recipients after AL or DR for 1 month, along with competitive BMCs from mice without transplantation at a 4:1 ratio, into AL-recipient mice greatly reduced the serially transplanted HSCs’ ability to compete for hematopoietic reconstruction compared with that of competing cells (the chimerism of donor-derived cells in peripheral blood was only 3.26% in the 2nd treatment and 0.92% in the 3rd treatment). This indicated that the HSCs’ potential for hematopoietic reconstruction was significantly reduced after transplantation. Interestingly, the chimerism was significantly higher in DR-donor-derived cells than in AL-donor-derived cells. These results indicate that short-term DR after transplantation significantly preserved and improved the regeneration capacity of transplanted HSCs. Mechanistically, the transplantation experiment with pure HSCs showed that the transplanted HSCs in the AL-recipient mice expanded by 304-fold at 3 weeks of transplantation, whereas they expanded only 17-fold in the DR recipients. These results further show that DR significantly inhibited HSC proliferation, even under the great pressure of hematopoietic reconstruction, which we speculate may be an important mechanism by which DR preserves HSC function.

The results of long-term (4 months) and short-term (1 month) DR in the secondary transplantation group differed. After transplanting BMCs from recipients 4 months after AL or DR at a 4:1 ratio with competitive BMCs from untransplanted mice into AL-recipient mice, the number of chimeric cells derived from AL mice was higher than that from mice that underwent serial transplantation 1 month after primary transplantation. At this timepoint, DR for 4 months after transplantation did not improve the chimerism compared with that of the AL mice. However, in the tertiary transplantation, DR-donor-derived chimerism was higher than was AL-donor-derived chimerism. Analysis of the BMCs harvested at 1 and 4 months post-transplantation suggested that serially transplanting the HSCs within 1 month impaired the reconstitution potential of the HSCs more strongly than did transplantation within 4 months, whereas DR did not appear to affect this. This may be because in addition to hematopoietic reconstruction pressure, HSCs are also affected by a strong inflammatory response in the recipient’s body early after transplantation, which may also harm the HSCs’ survival and functioning. We and others have reported that DR can reduce inflammation; therefore, it may protect HSCs by inhibiting proliferation and inflammation at earlier times after transplantation, which requires further study. Previous studies have shown that in the early stages post-HSCT, patients release large amounts of inflammatory cytokines [29–31], and HSCs can sense inflammatory stimuli, proliferating to produce more defense cells [32]. Interestingly, we and others have reported that DR can reduce inflammation [27, 28]. Therefore, we speculate that post-transplant DR in the early stages reduces inflammation, thus reducing HSC proliferation and maintaining HSC function. This hypothesis requires further research.

At present, in situ bone tissue engineering is garnering significant interest. For example, Krasilnikova et al. have reported for repairing bone tissue defects by isolating autologous osteoblasts, bone marrow stromal cells, or chondrocytes, culturing and expanding them in vitro, then seeding them onto synthetic scaffolds, and implanting them into bone defect sites [33, 34]. Our research suggests that DR during hematopoietic system regeneration can inhibit the repair of the bone marrow microenvironment and impair HSC reconstruction, thereby significantly delaying hematopoietic recovery. Whether similar phenotypes can be replicated in other regenerative medicine models is a very interesting question that could have important implications for improving the regeneration of corresponding tissues. This will require further research to confirm.

In summary, our results suggest that even under the great pressure of hematopoietic reconstruction, DR significantly inhibited HSC expansion, which protected the reconstitution capacity of HSCs. However, DR also suppressed and delayed post-transplant hematopoiesis. Because reduced food intake and problems with digestion and absorption are common in patients undergoing HCT, our findings show that caloric management after transplantation deserves attention.

## Electronic Supplementary Material

Below is the link to the electronic supplementary material.

Supplementary Material 1

## Data Availability

The datasets used and analyzed during the current study are available from thecorresponding author on reasonable request.
