# Supplementary material for: Effect of Post-transplant Dietary Restriction on Hematopoietic Reconstitution and Maintenance of Reconstitution Capacity of Hematopoietic Stem Cells
Source: Stem Cell Rev Rep. 2024 Jul 5;21(1):80–95. doi: 10.1007/s12015-024-10754-y (PMC11762425; doi:10.1007/s12015-024-10754-y)
Supplement: Supplementary file 1 — Supplementary Material 1 [file 12015_2024_10754_MOESM1_ESM.pdf]

Supplemental meterial

1 month AL

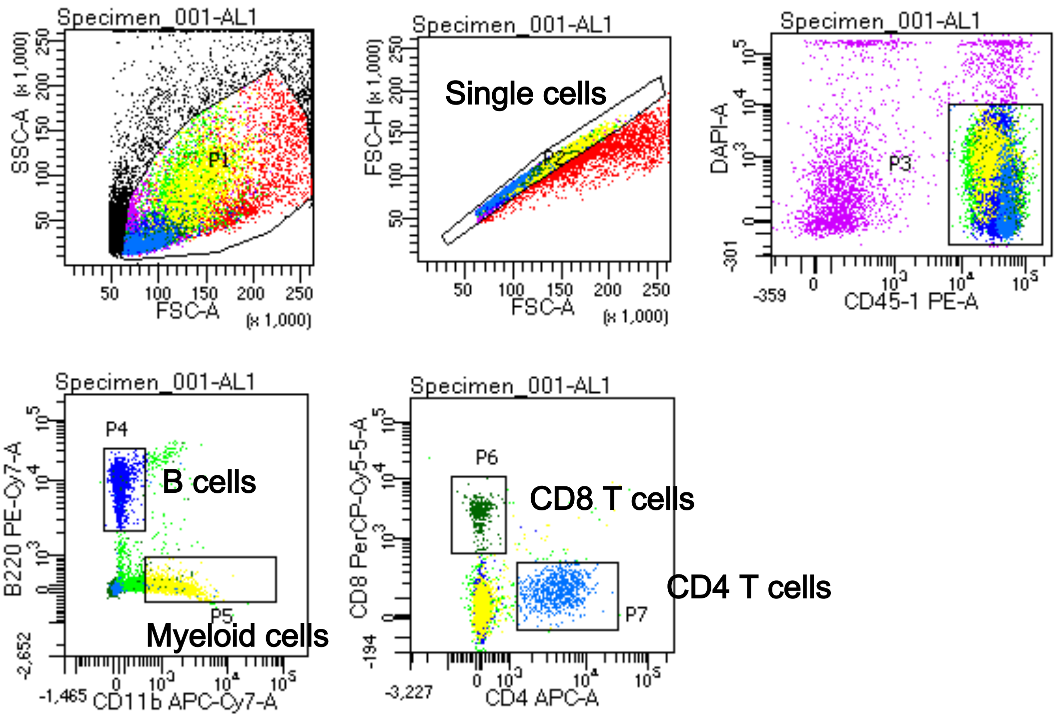

Tube: AL1

Population	#Events	%Parent	%Total
All Events	21,176	####	100.0
P1	13,741	64.9	64.9
P2	11,559	84.1	54.6
P3	9,053	78.3	42.8
P4	2,851	31.5	13.5
P5	2,276	25.1	10.7
P6	369	4.1	1.7
P7	757	8.4	3.6

Fig. S1a Gating strategy of B/T/Myeloid cell in PB in flow cytometry analysis.

1 month DR

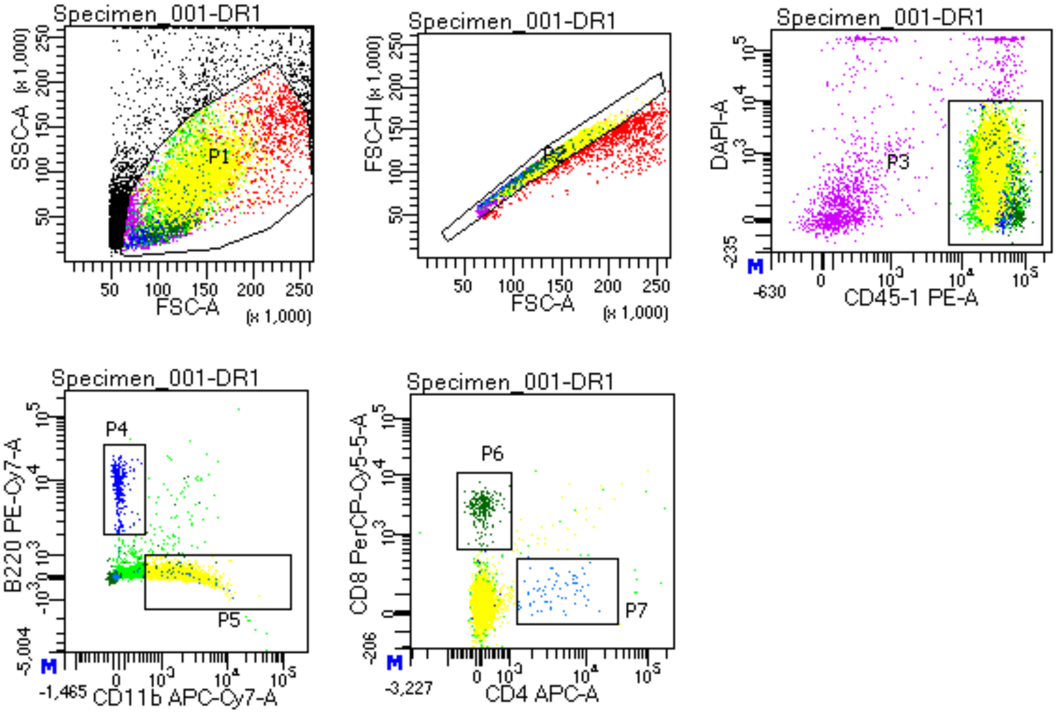

Tube: DR1

Population	#Events	%Parent	%Total
All Events	16,645	####	100.0
P1	11,344	68.2	68.2
P2	10,303	90.8	61.9
P3	9,005	87.4	54.1
P4	277	3.1	1.7
P5	5,903	65.6	35.5
P6	238	2.6	1.4
P7	71	0.8	0.4

Fig. S1b Gating strategy of B/T/Myeloid cell in PB in flow cytometry analysis.

4 months AL

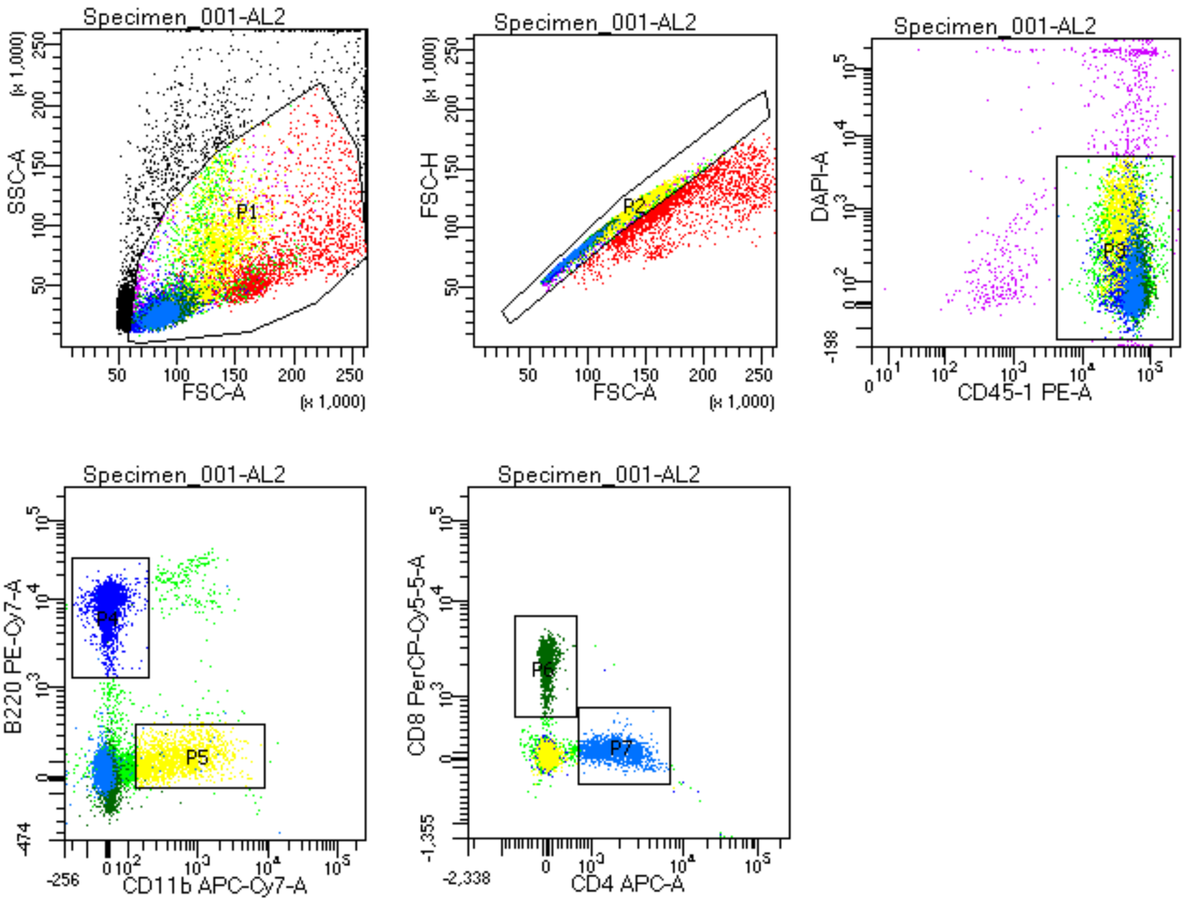

Tube: AL2

Population	#Events	%Parent	%Total
All Events	16,349	####	100.0
P1	12,476	76.3	76.3
P2	10,336	82.8	63.2
P3	9,695	93.8	59.3
P4	3,367	34.7	20.6
P5	1,602	16.5	9.8
P6	1,411	14.6	8.6
P7	1,363	14.1	8.3

Fig. S1c Gating strategy of B/T/Myeloid cell in PB in flow cytometry analysis.

4 months DR

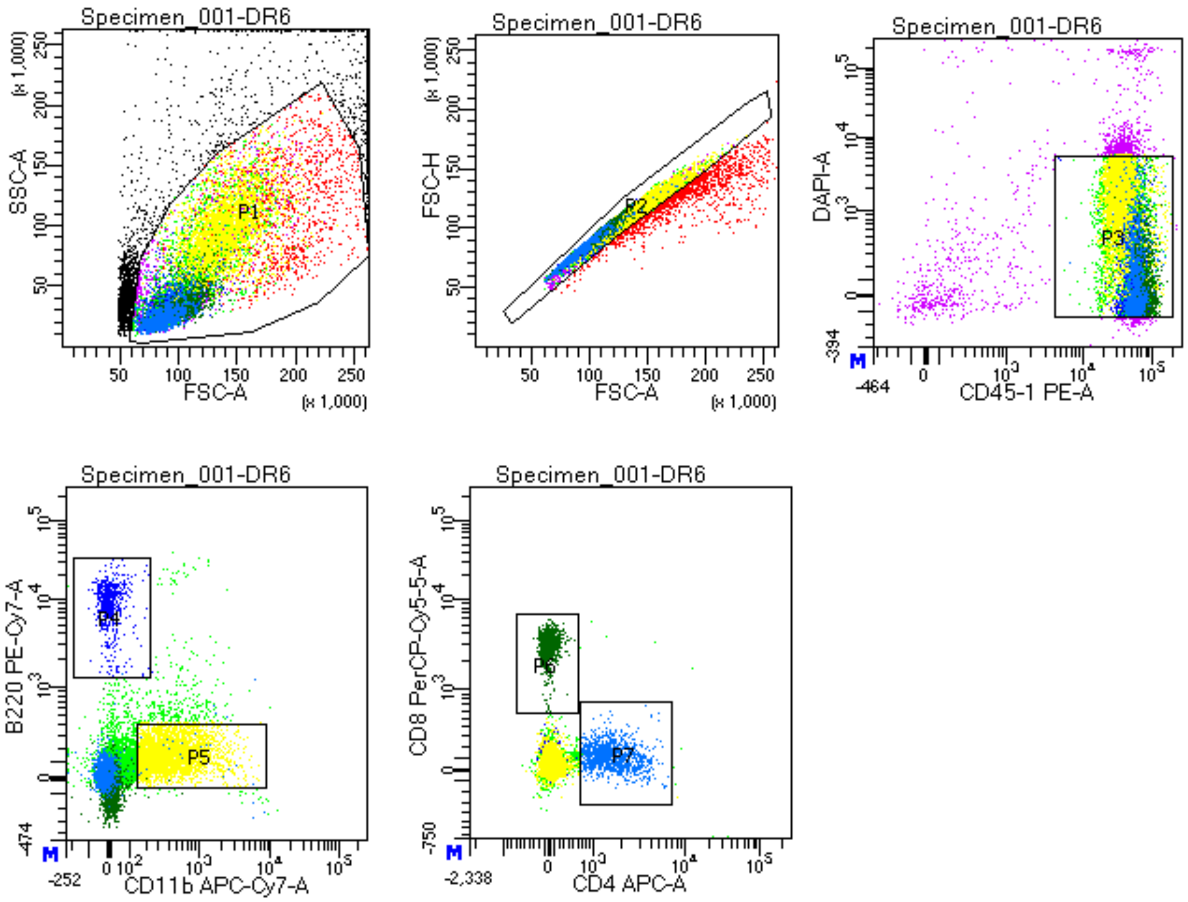

Tube: DR6

Population	#Events	%Parent	%Total
All Events	14,770	####	100.0
P1	11,600	78.5	78.5
P2	10,240	88.3	69.3
P3	9,138	89.2	61.9
P4	699	7.6	4.7
P5	3,182	34.8	21.5
P6	1,205	13.2	8.2
P7	1,078	11.8	7.3

Fig. S1d Gating strategy of B/T/Myeloid cell in PB in flow cytometry analysis.

1 month AL

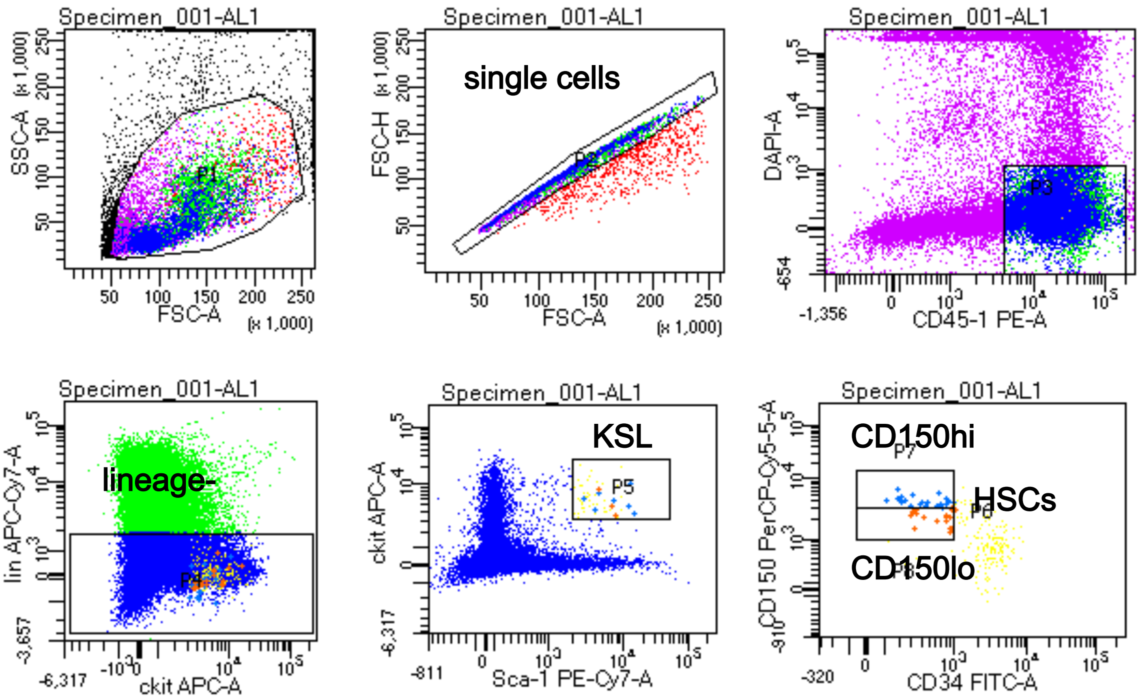

Tube: AL1

Population	#Events	%Parent	%Total
All Events	1,000,000	####	100.0
P1	772,677	77.3	77.3
P2	703,916	91.1	70.4
P3	386,242	54.9	38.6
P4	187,157	48.5	18.7
P5	213	0.1	0.0
P6	41	19.2	0.0
P7	25	61.0	0.0
P8	17	41.5	0.0

Fig. S2a Gating strategy of HSCs in flow cytometry analysis.

1 month DR

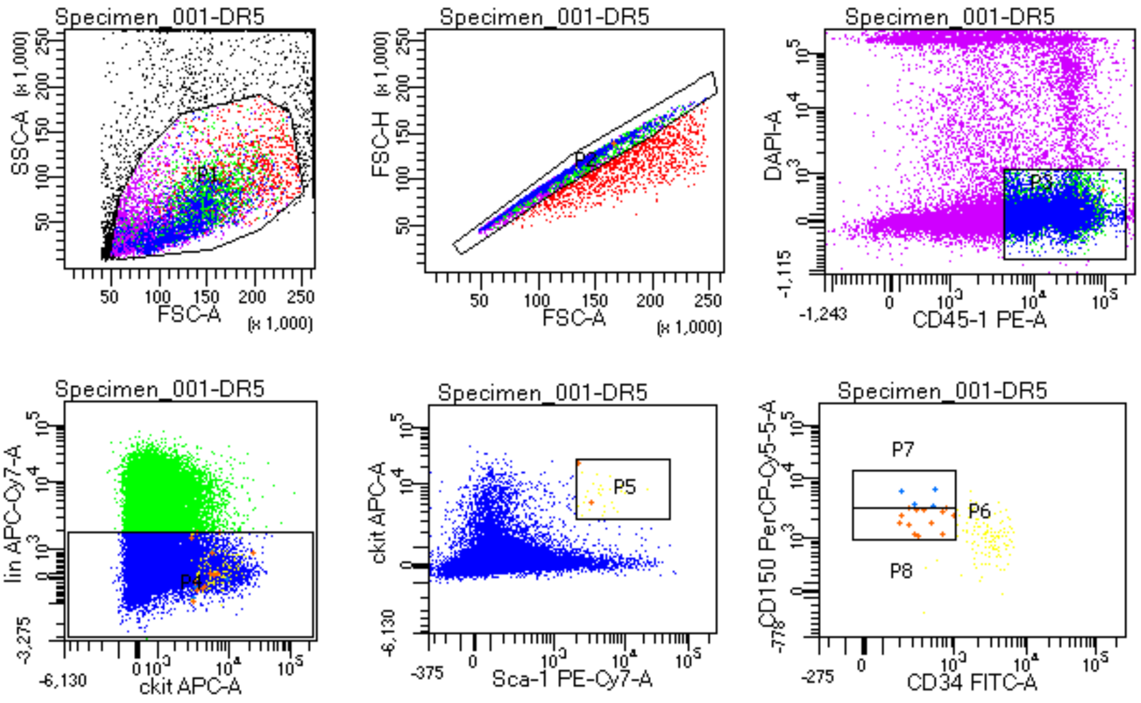

Tube: DR5

Population	#Events	%Parent	%Total
All Events	1,000,000	####	100.0
P1	707,103	70.7	70.7
P2	587,164	83.0	58.7
P3	280,278	47.7	28.0
P4	141,627	50.5	14.2
P5	143	0.1	0.0
P6	17	11.9	0.0
P7	5	29.4	0.0
P8	13	76.5	0.0

Fig. S2b Gating strategy of HSCs in flow cytometry analysis.

4 months AL

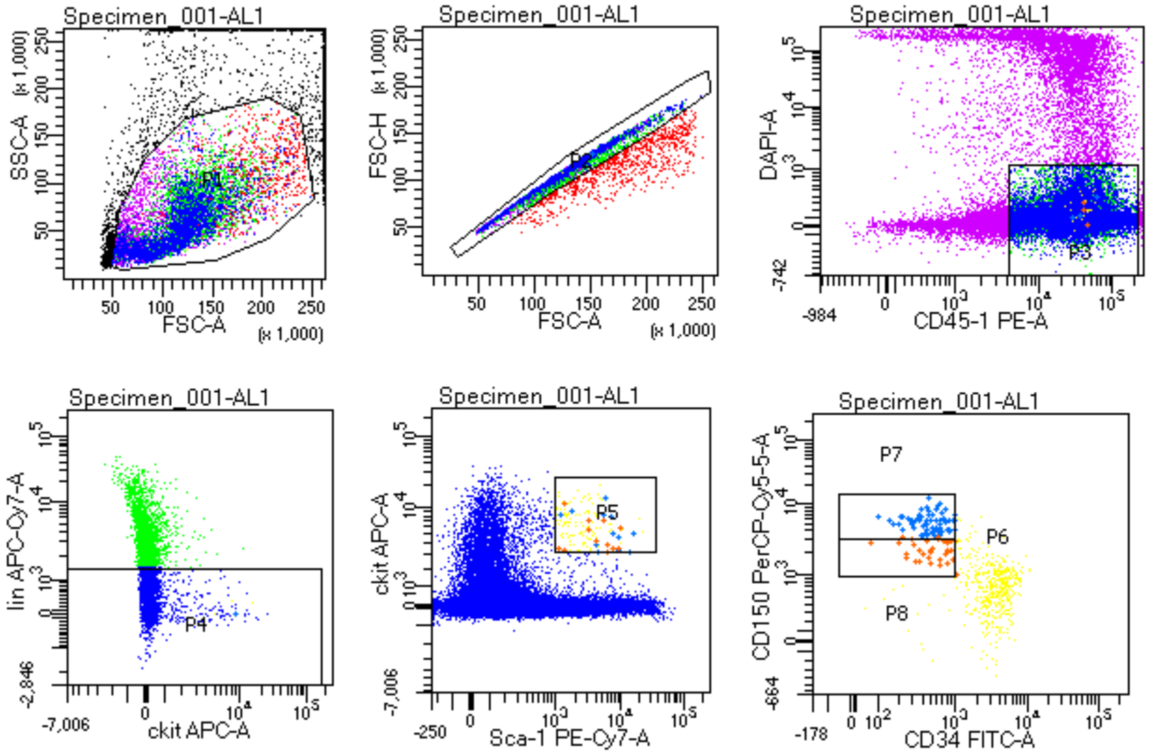

Tube: AL1

Population	#Events	%Parent	%Total
All Events	1,000,000	####	100.0
P1	781,139	78.1	78.1
P2	694,592	88.9	69.5
P3	500,594	72.1	50.1
P4	248,560	49.7	24.9
P5	594	0.2	0.1
P6	101	17.0	0.0
P7	70	69.3	0.0
P8	33	32.7	0.0

Fig. S2c Gating strategy of HSCs in flow cytometry analysis.

4 months DR

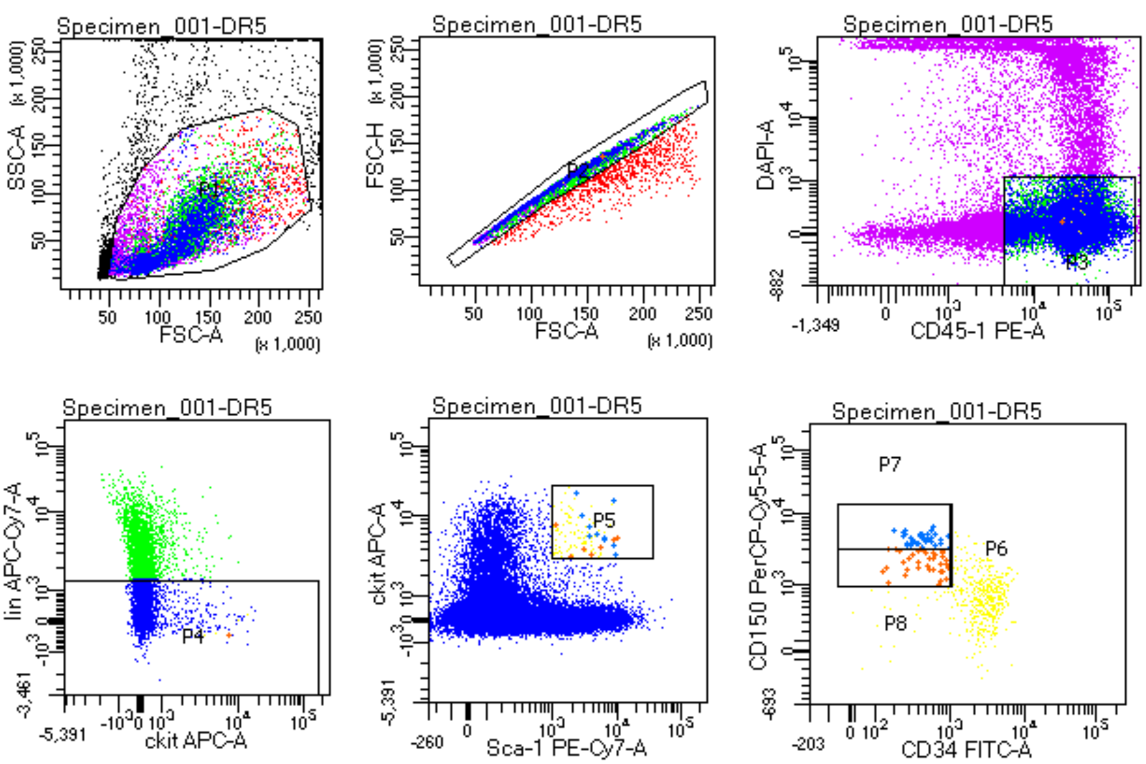

Tube: DR5

Population	#Events	%Parent	%Total
All Events	1,000,000	####	100.0
P1	776,805	77.7	77.7
P2	687,249	88.5	68.7
P3	462,827	67.3	46.3
P4	219,376	47.4	21.9
P5	536	0.2	0.1
P6	77	14.4	0.0
P7	39	50.6	0.0
P8	41	53.2	0.0

Fig. S2d Gating strategy of HSCs in flow cytometry analysis.

1 month AL

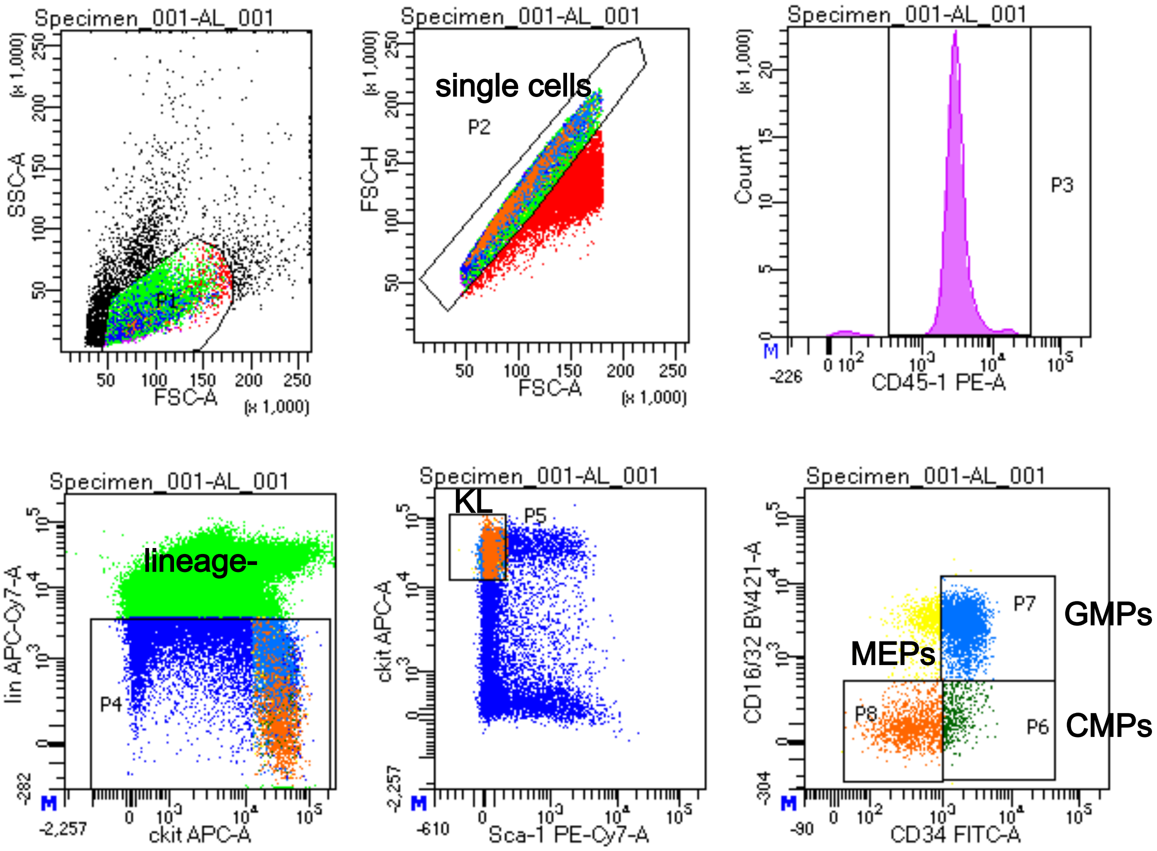

Tube: AL_001

Population	#Events	%Parent	%Total
All Events	500,000	####	100.0
P1	443,883	88.8	88.8
P2	434,434	97.9	86.9
P3	415,873	95.7	83.2
P4	16,020	3.9	3.2
P5	5,068	31.6	1.0
P6	420	8.3	0.1
P7	2,764	54.5	0.6
P8	1,210	23.9	0.2

Fig. S2e Gating strategy of CMPs/GMPs/MEPs in flow cytometry analysis.

1 month DR

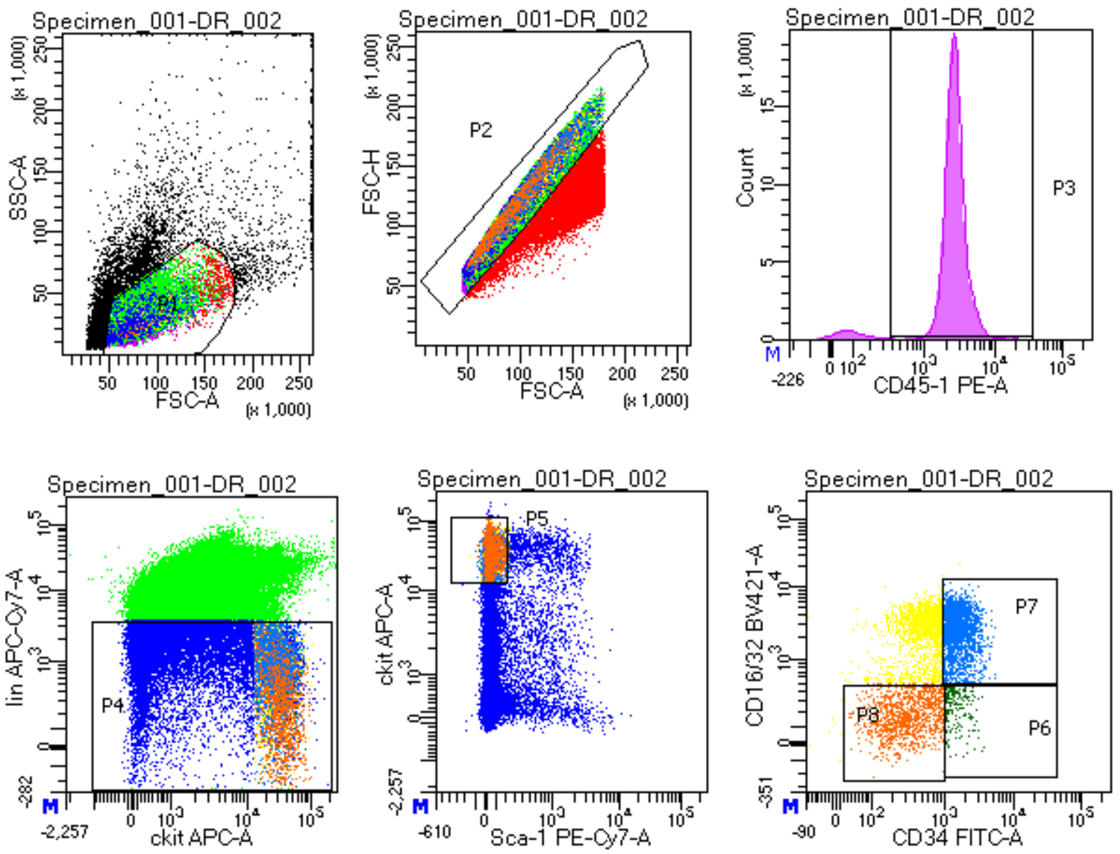

Tube: DR_002

Population	#Events	%Parent	%Total
All Events	500,000	####	100.0
P1	409,516	81.9	81.9
P2	395,874	96.7	79.2
P3	367,741	92.9	73.5
P4	24,142	6.6	4.8
P5	5,670	23.5	1.1
P6	212	3.7	0.0
P7	2,384	42.0	0.5
P8	1,196	21.1	0.2

Fig. S2f Gating strategy of CMPs/GMPs/MEPs in flow cytometry analysis.

4 months AL

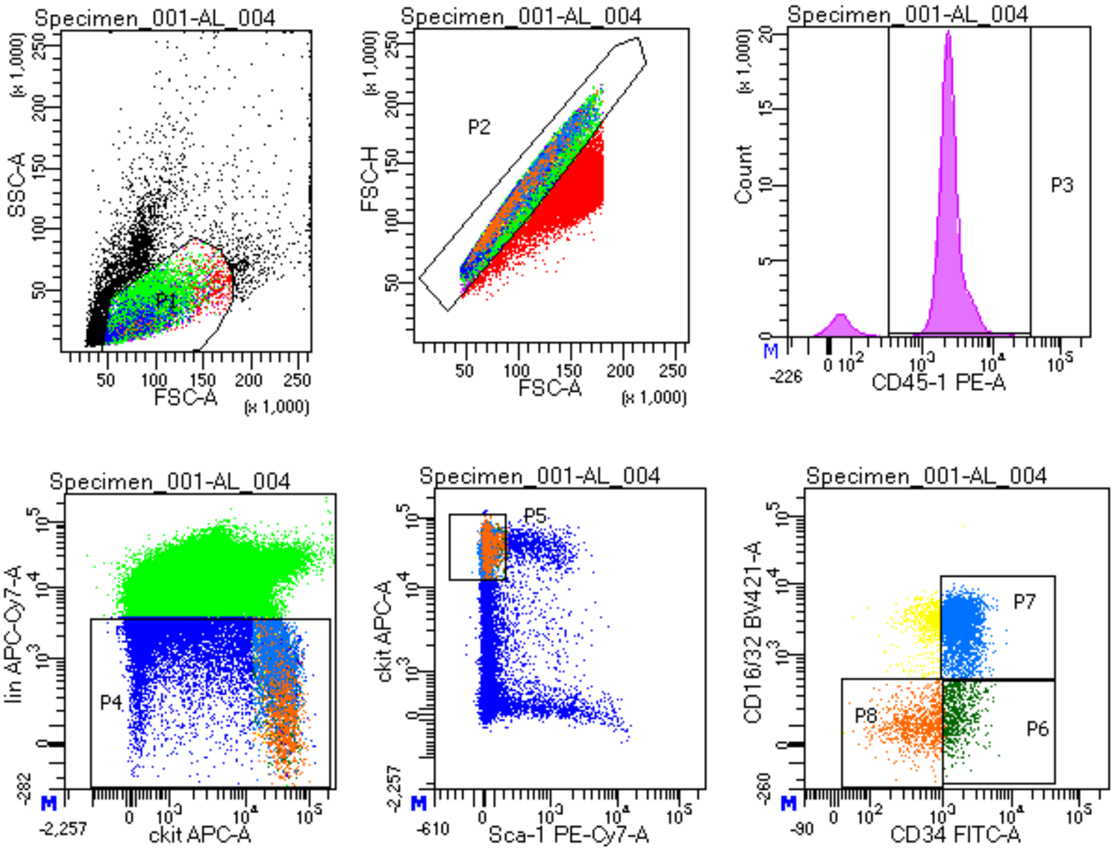

Tube: AL_004

Population	#Events	%Parent	%Total
All Events	500,000	####	100.0
P1	426,528	85.3	85.3
P2	416,445	97.6	83.3
P3	373,989	89.8	74.8
P4	16,667	4.5	3.3
P5	4,915	29.5	1.0
P6	633	12.9	0.1
P7	2,785	56.7	0.6
P8	922	18.8	0.2

Fig. S2g Gating strategy of CMPs/GMPs/MEPs in flow cytometry analysis

4 months DR

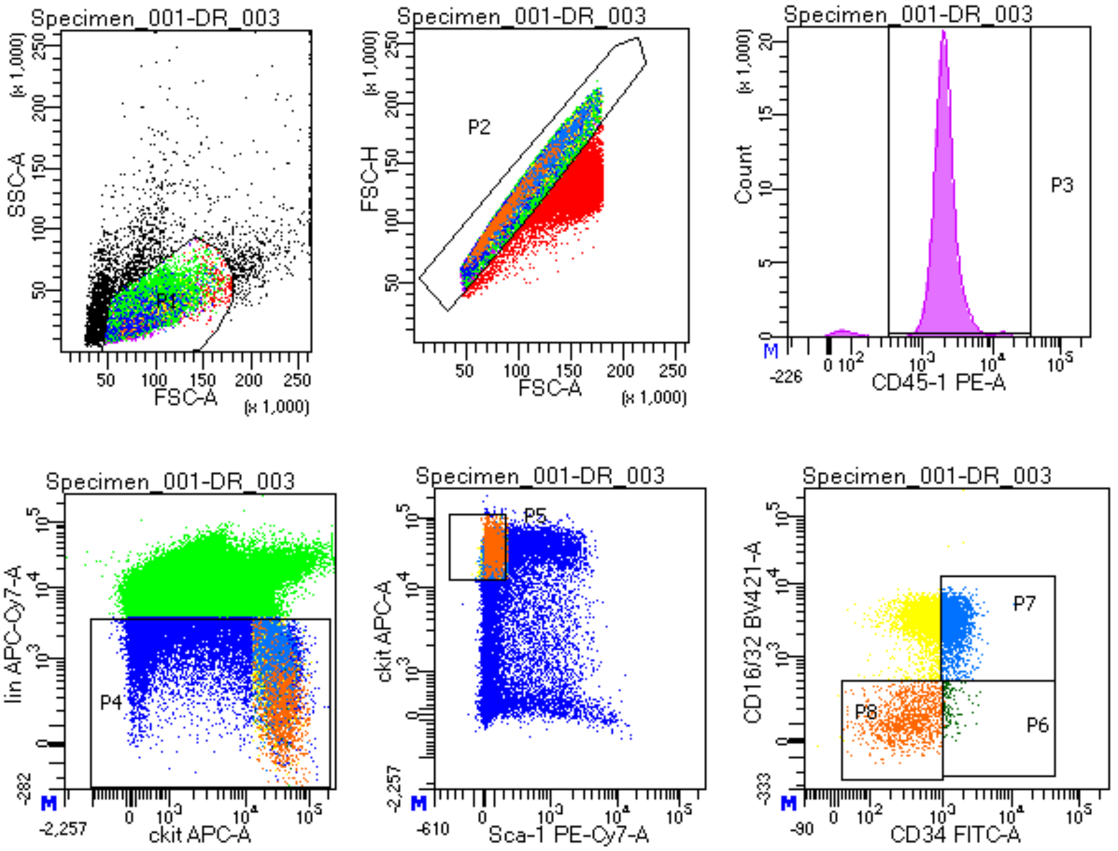

Tube: DR_003

Population	#Events	%Parent	%Total
All Events	500,000	####	100.0
P1	441,567	88.3	88.3
P2	434,283	98.4	86.9
P3	417,076	96.0	83.4
P4	22,185	5.3	4.4
P5	6,278	28.3	1.3
P6	125	2.0	0.0
P7	2,265	36.1	0.5
P8	1,124	17.9	0.2

Fig. S2h Gating strategy of CMPs/GMPs/MEPs in flow cytometry analysis.

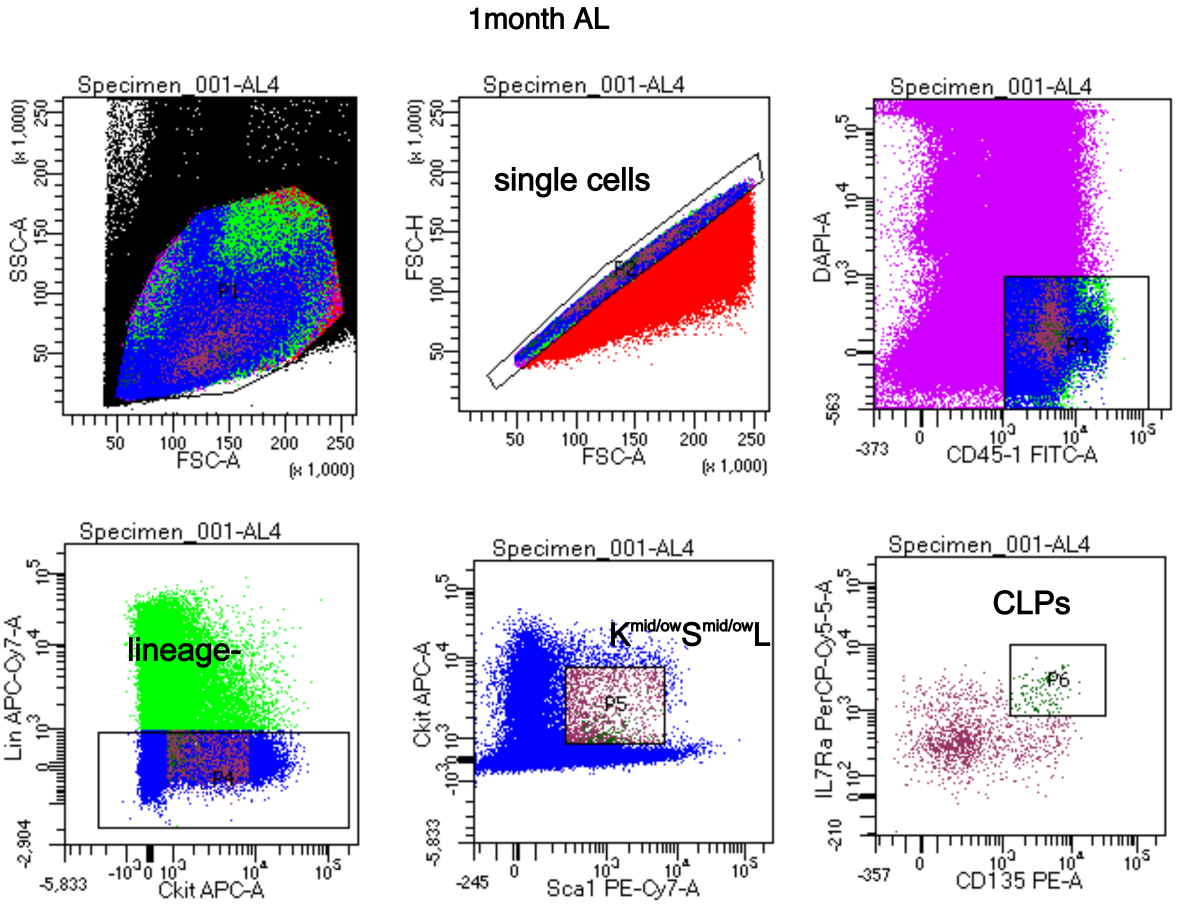

Tube: AL4

Population	#Events	%Parent	%Total
All Events	1,000,000	####	100.0
P1	752,898	75.3	75.3
P2	654,357	86.9	65.4
P3	309,233	47.3	30.9
P4	116,410	37.6	11.6
P5	1,373	1.2	0.1
P6	122	8.9	0.0

Fig. S2i Gating strategy of CLPs in flow cytometry analysis.

1month DR

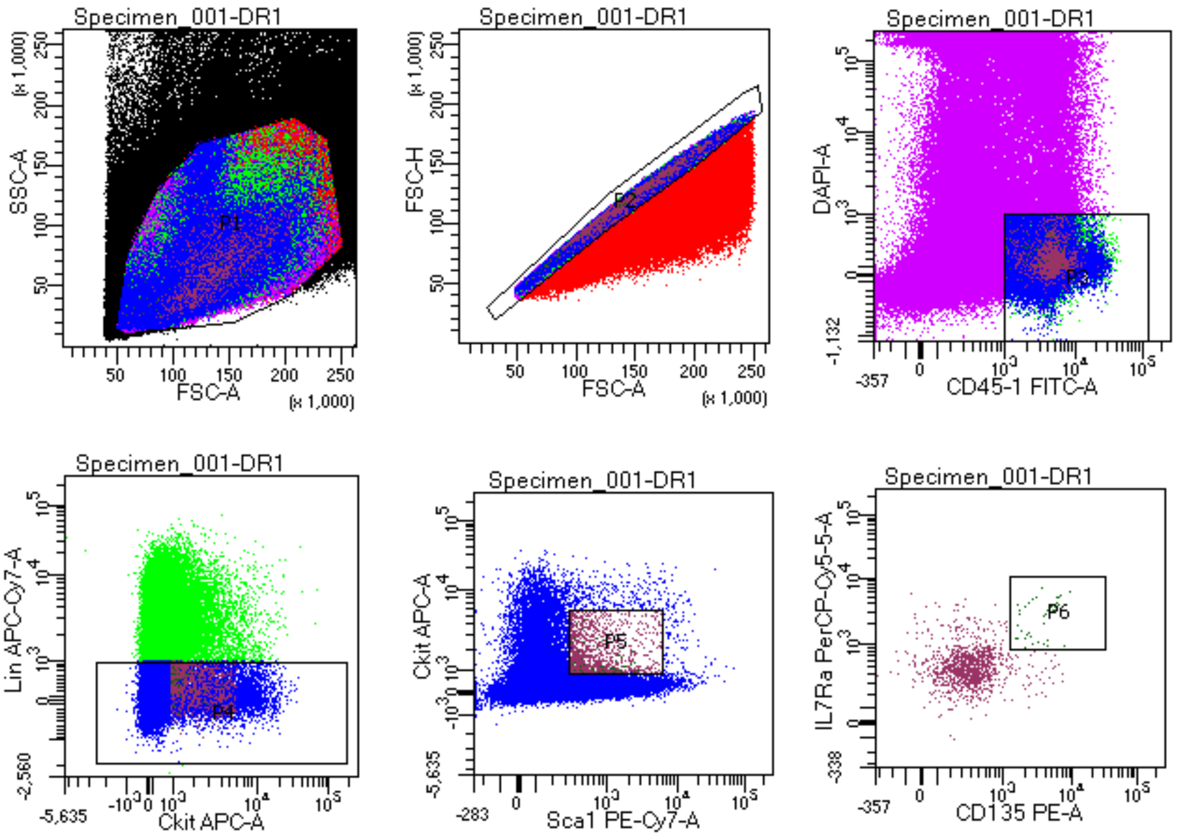

Tube: DR1

Population	#Events	%Parent	%Total
All Events	1,000,000	####	100.0
P1	749,169	74.9	74.9
P2	596,861	79.7	59.7
P3	163,687	27.4	16.4
P4	74,651	45.6	7.5
P5	1,137	1.5	0.1
P6	44	3.9	0.0

Fig. S2j Gating strategy of CLPs in flow cytometry analysis.

4 months AL

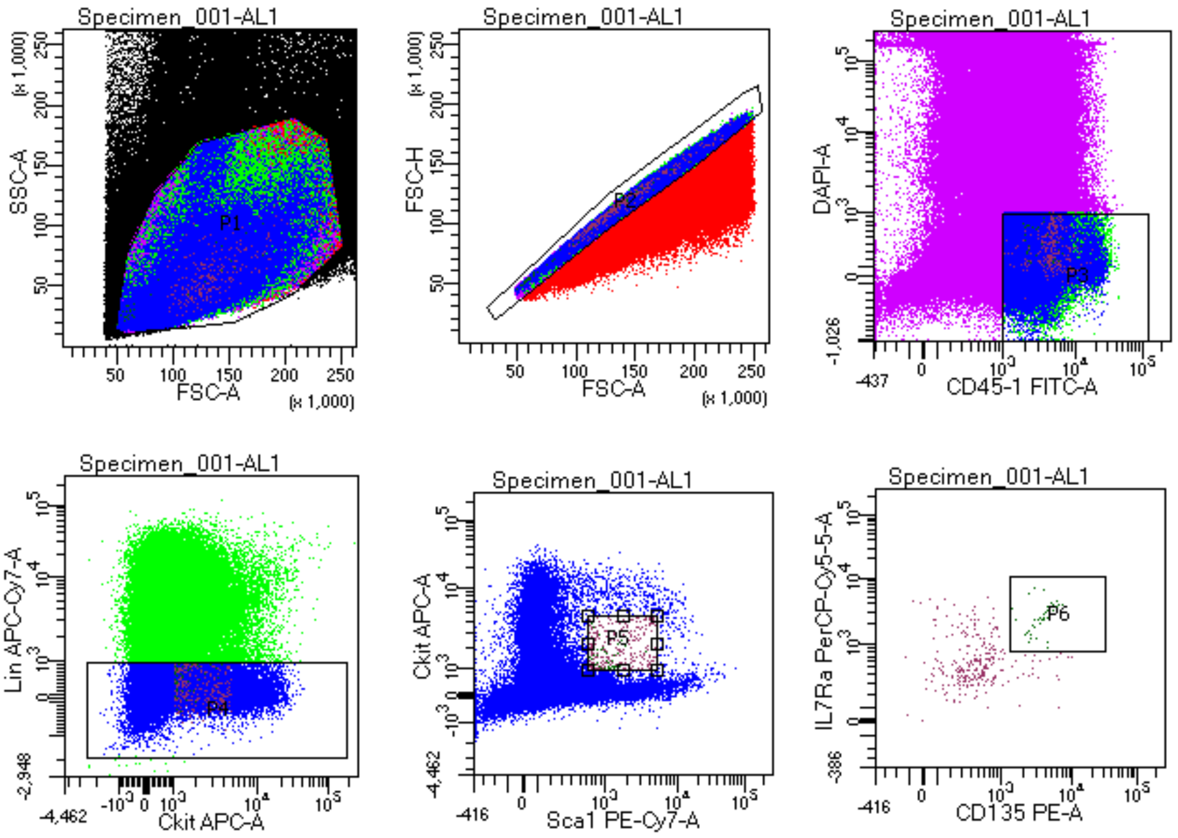

Tube: AL1

Population	#Events	%Parent	%Total
All Events	1,000,000	####	100.0
P1	773,645	77.4	77.4
P2	693,574	89.7	69.4
P3	395,816	57.1	39.6
P4	118,757	30.0	11.9
P5	305	0.3	0.0
P6	41	13.4	0.0

Fig. S2k Gating strategy of CLPs in flow cytometry analysis.

4 months DR

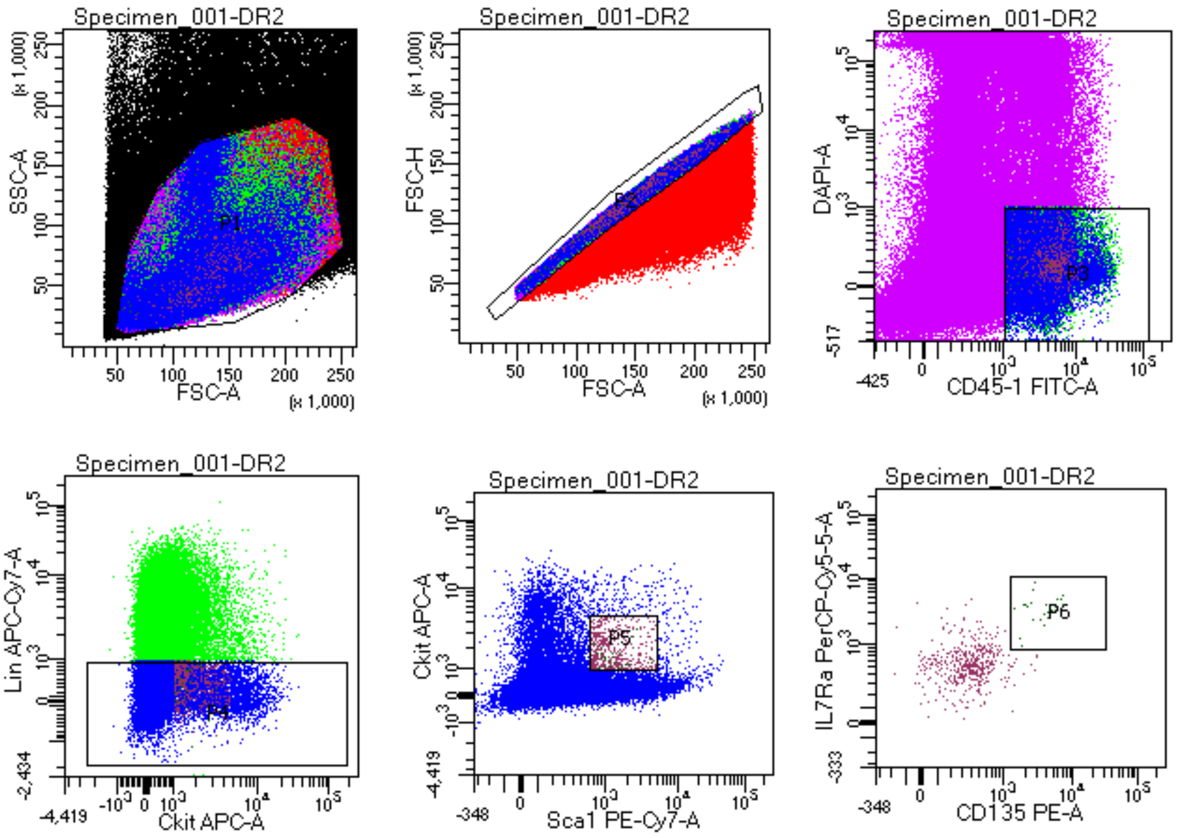

Tube: DR2

Population	#Events	%Parent	%Total
All Events	1,000,000	####	100.0
P1	729,152	72.9	72.9
P2	588,667	80.7	58.9
P3	112,042	19.0	11.2
P4	49,446	44.1	4.9
P5	478	1.0	0.0
P6	25	5.2	0.0

Fig. S2I Gating strategy of CLPs in flow cytometry analysis.

1 month AL

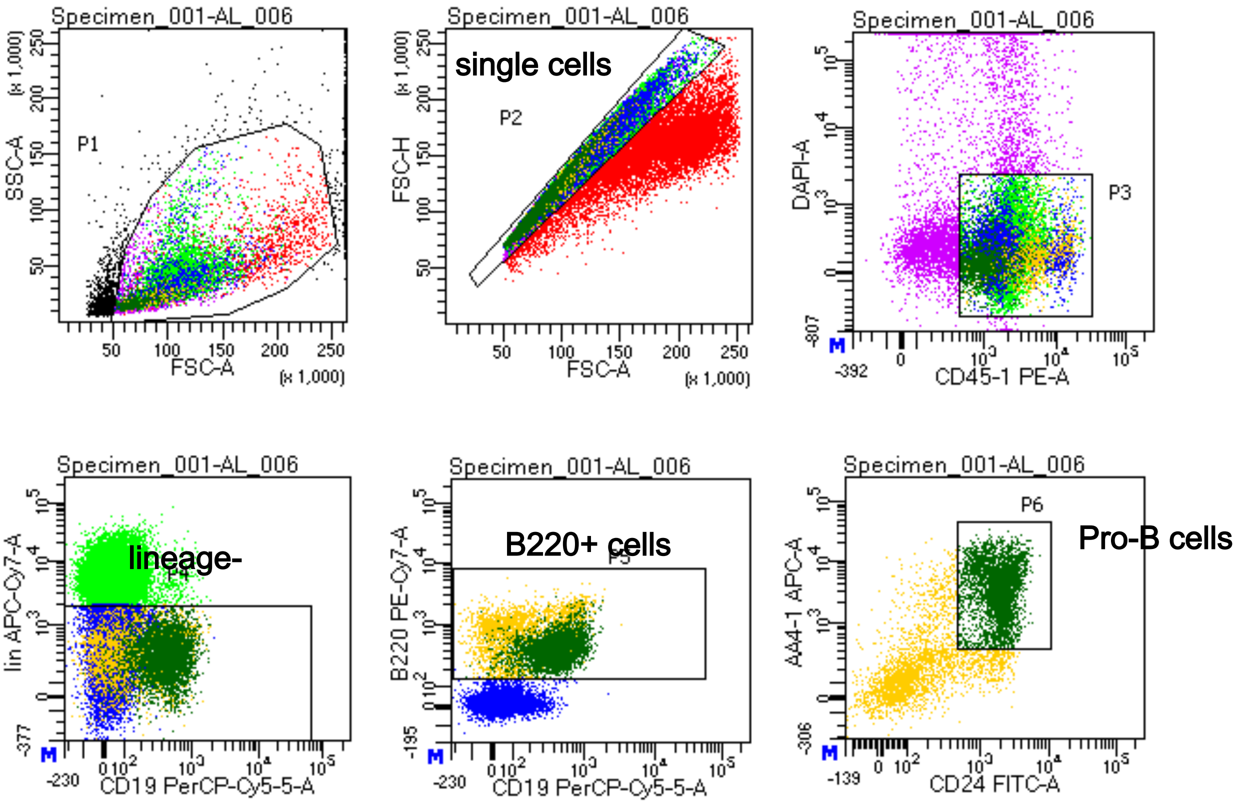

Tube: AL_006

Population	#Events	%Parent	%Total
All Events	100,000	####	100.0
P1	84,255	84.3	84.3
P2	73,381	87.1	73.4
P3	60,949	83.1	60.9
P4	15,722	25.8	15.7
P5	6,338	40.3	6.3
P6	3,764	59.4	3.8

Fig. S2m Gating strategy of Pro-B cell in flow cytometry analysis.

1 month DR

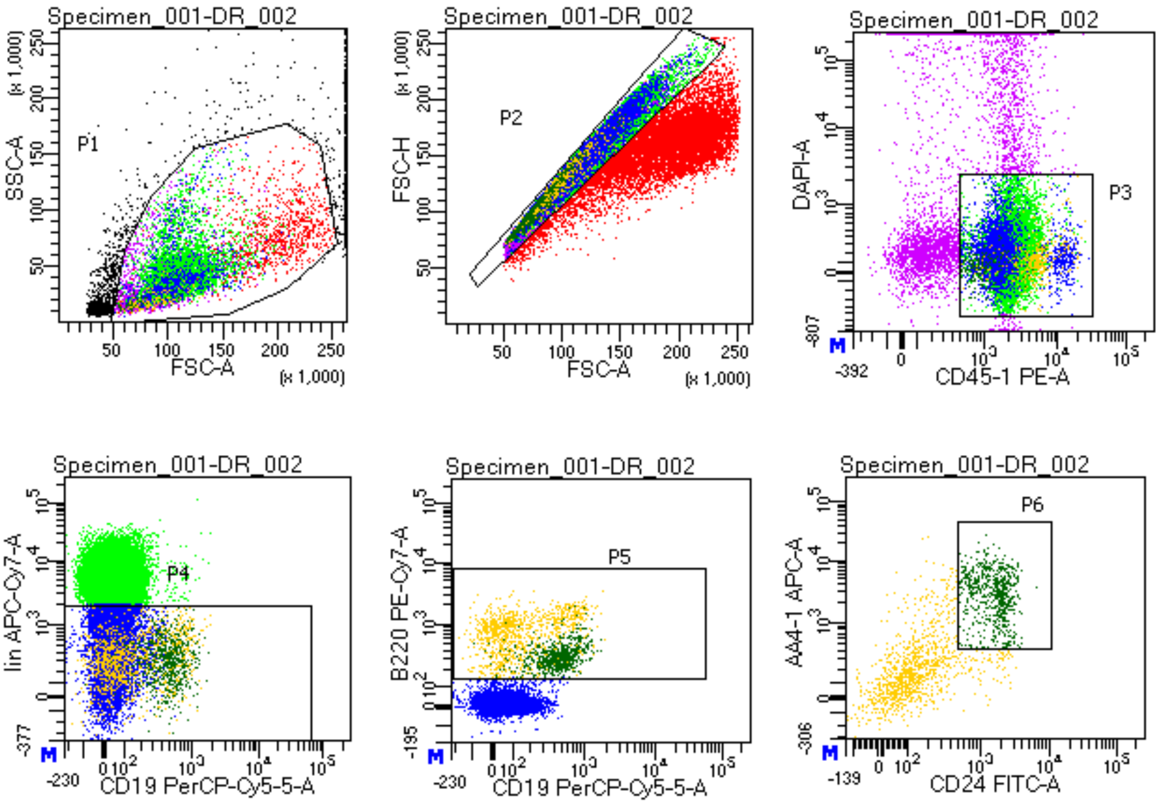

Tube: DR_002

Population	#Events	%Parent	%Total
All Events	100,000	####	100.0
P1	87,320	87.3	87.3
P2	77,382	88.6	77.4
P3	67,022	86.6	67.0
P4	9,822	14.7	9.8
P5	1,682	17.1	1.7
P6	632	37.6	0.6

Fig. S2n Gating strategy of Pro-B cell in flow cytometry analysis.

4 months AL

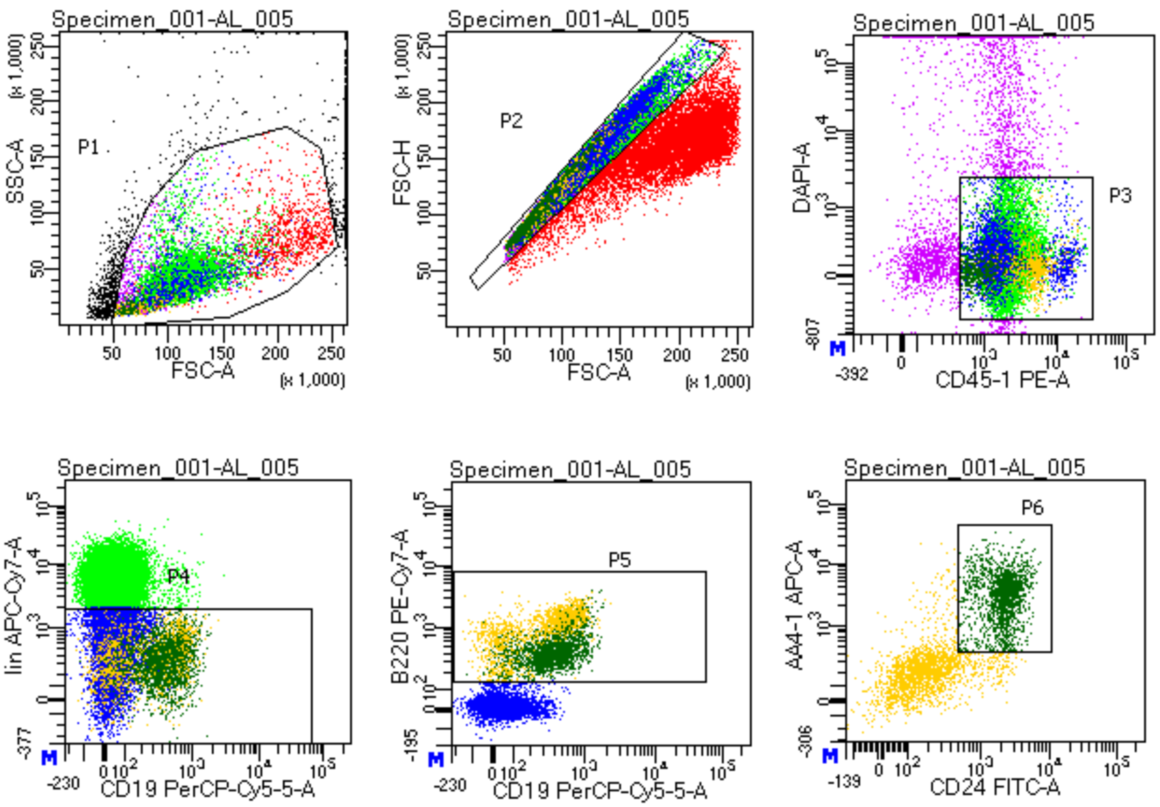

Tube: AL_005

Population	#Events	%Parent	%Total
All Events	100,000	####	100.0
P1	87,310	87.3	87.3
P2	76,805	88.0	76.8
P3	68,803	89.6	68.8
P4	11,009	16.0	11.0
P5	3,588	32.6	3.6
P6	1,620	45.2	1.6

Fig. S2o Gating strategy of Pro-B cells in flow cytometry analysis.

4 months DR

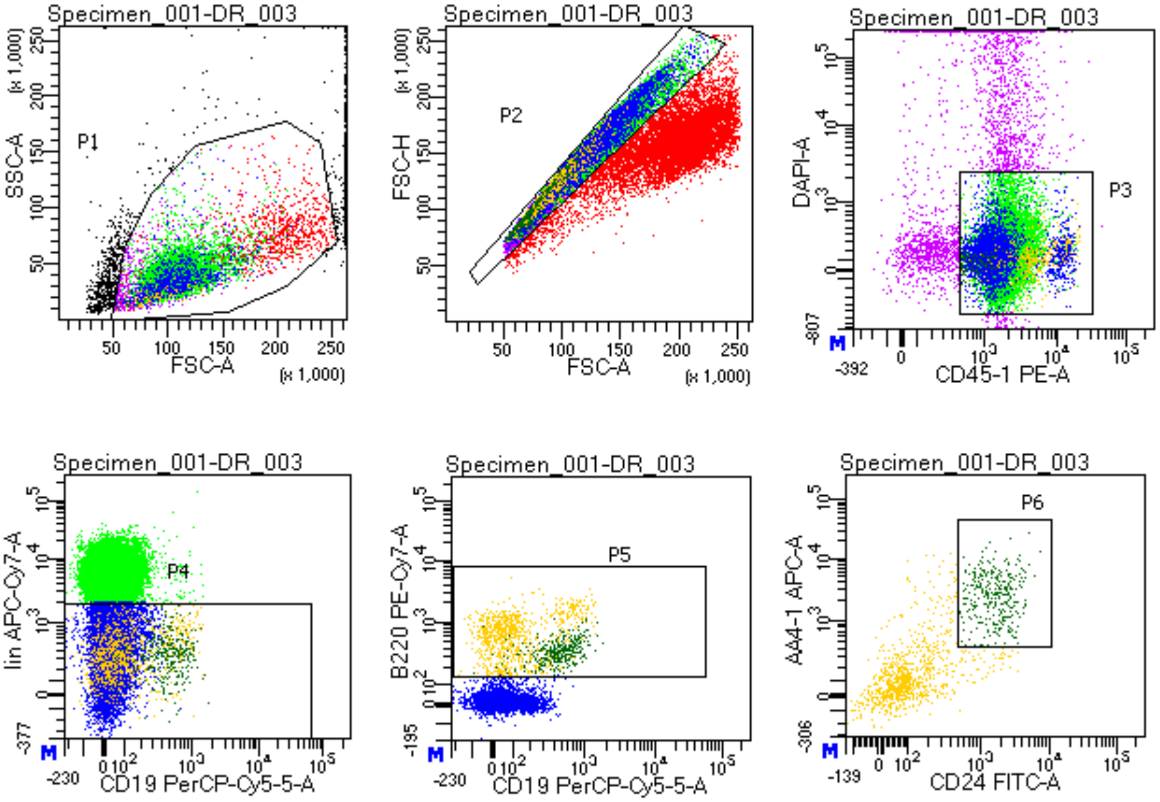

Tube: DR_003

Population	#Events	%Parent	%Total
All Events	100,000	####	100.0
P1	90,217	90.2	90.2
P2	81,387	90.2	81.4
P3	74,081	91.0	74.1
P4	10,798	14.6	10.8
P5	1,194	11.1	1.2
P6	317	26.5	0.3

Fig. S2p Gating strategy of Pro-B cells in flow cytometry analysis.

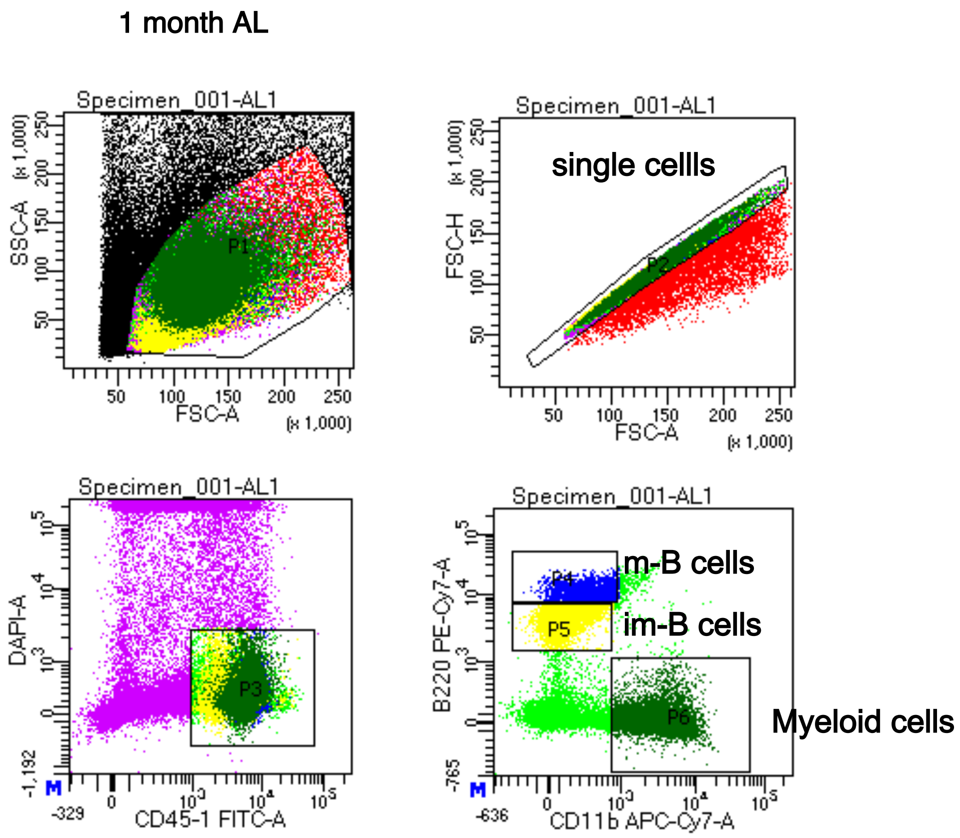

Tube: AL1

Population	#Events	%Parent	%Total
All Events	120,744	####	100.0
P1	83,932	69.5	69.5
P2	76,403	91.0	63.3
P3	42,440	55.5	35.1
P4	1,899	4.5	1.6
P5	9,341	22.0	7.7
P6	22,875	53.9	18.9

Fig. S2q Gating strategy of B/Myeloid cell in BM in flow cytometry analysis.

1 month DR

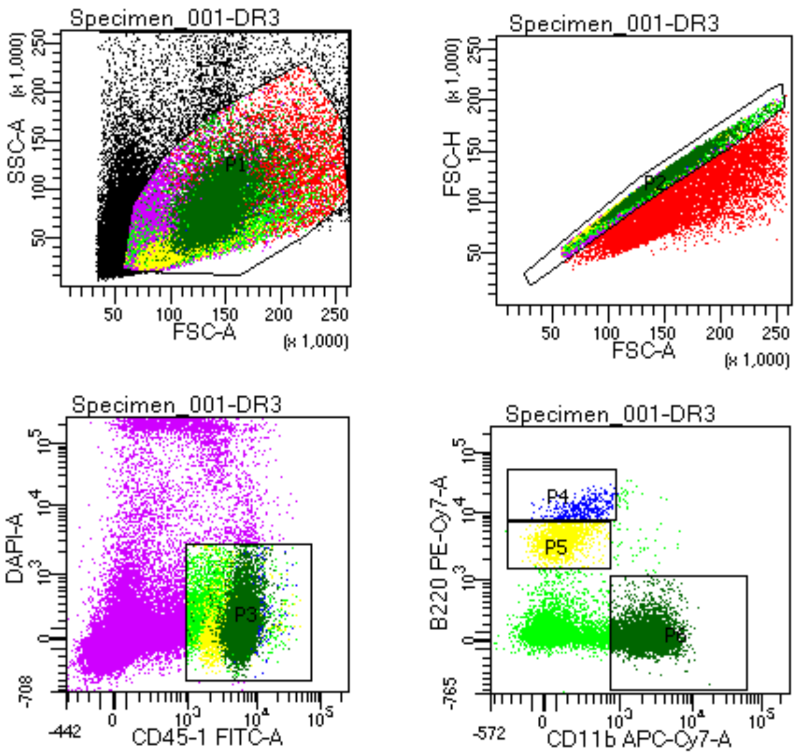

Tube: DR3

Population	#Events	%Parent	%Total
All Events	93,853	####	100.0
P1	63,902	68.1	68.1
P2	54,320	85.0	57.9
P3	14,529	26.7	15.5
P4	338	2.3	0.4
P5	1,491	10.3	1.6
P6	7,912	54.5	8.4

Fig. S2r Gating strategy of B/Myeloid cell in BM in flow cytometry analysis.

4 months AL

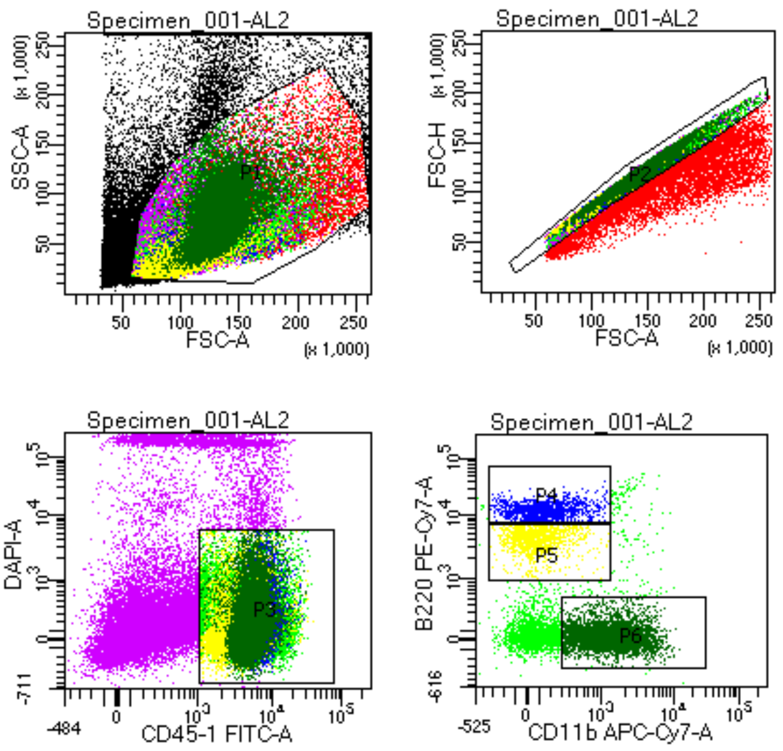

Tube: AL2

Population	#Events	%Parent	%Total
All Events	80,058	####	100.0
P1	53,850	67.3	67.3
P2	46,220	85.8	57.7
P3	27,908	60.4	34.9
P4	3,420	12.3	4.3
P5	3,776	13.5	4.7
P6	13,787	49.4	17.2

Fig. S2s Gating strategy of B/Myeloid cell in BM in flow cytometry analysis.

4 months DR

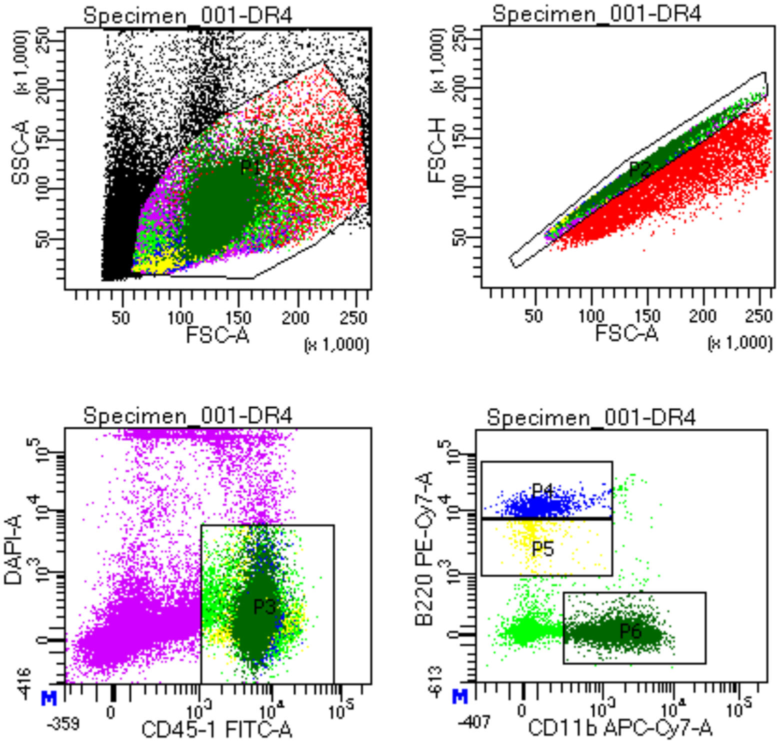

Tube: DR4

Population	#Events	%Parent	%Total
All Events	99,519	####	100.0
P1	60,404	60.7	60.7
P2	53,691	88.9	54.0
P3	26,383	49.1	26.5
P4	3,032	11.5	3.0
P5	970	3.7	1.0
P6	16,508	62.6	16.6

Fig. S2t Gating strategy of B/Myeloid cell in BM in flow cytometry analysis.

1 month (3rd Tx) AL

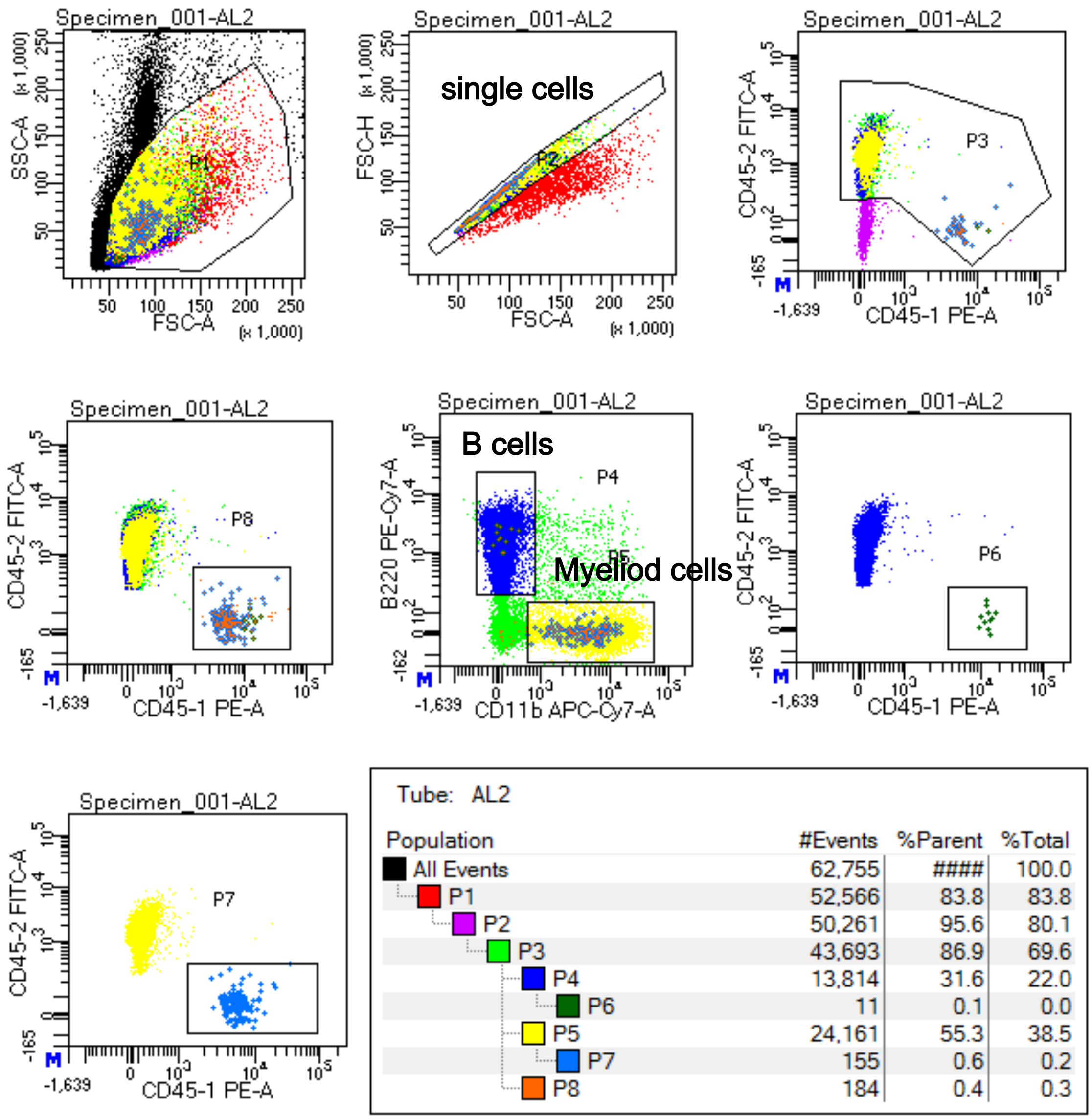

Population	#Events	%Parent	%Total
All Events	62,755	####	100.0
P1	52,566	83.8	83.8
P2	50,261	95.6	80.1
P3	43,693	86.9	69.6
P4	13,814	31.6	22.0
P6	11	0.1	0.0
P5	24,161	55.3	38.5
P7	155	0.6	0.2
P8	184	0.4	0.3

Fig. S3a Gating strategy of B/Myeloid cell in BM in flow cytometry analysis.

1 month (3rd Tx) DR

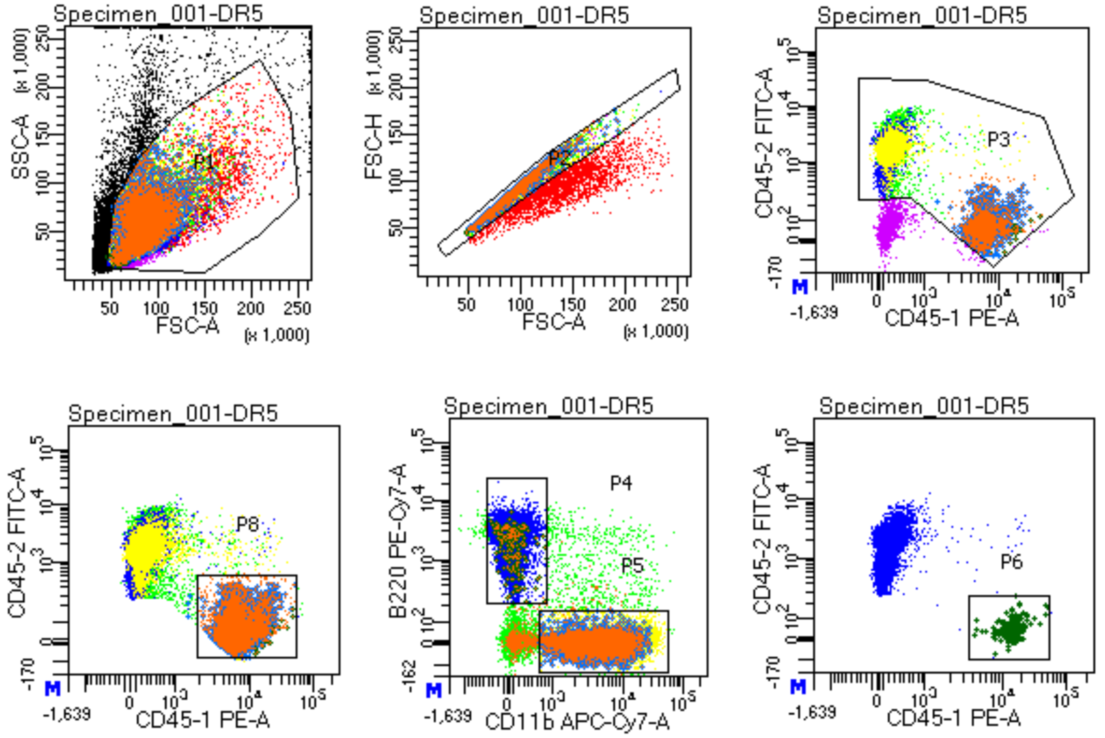

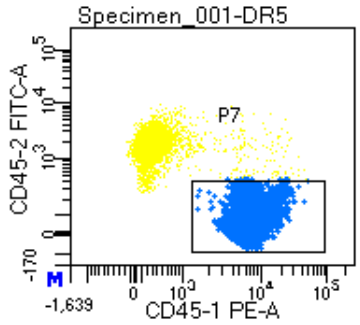

Tube: DR5

Population	#Events	%Parent	%Total
All Events	63,727	####	100.0
P1	54,001	84.7	84.7
P2	51,511	95.4	80.8
P3	43,646	84.7	68.5
P4	13,812	31.6	21.7
P6	202	1.5	0.3
P5	23,758	54.4	37.3
P7	6,878	29.0	10.8
P8	7,716	17.7	12.1

Fig. S3b Gating strategy of B/Myeloid cell in BM in flow cytometry analysis.

1 month (3rd Tx) AL

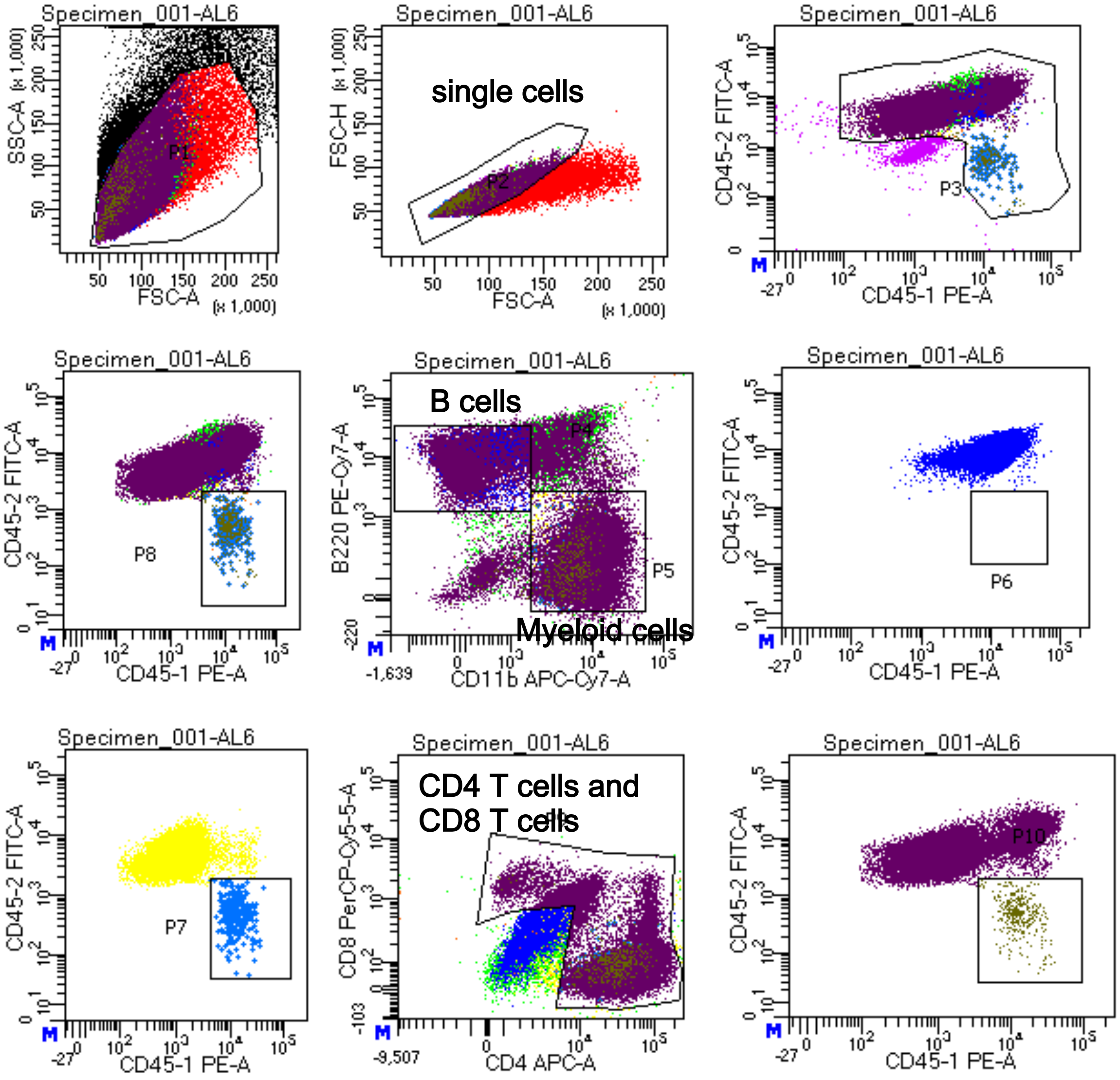

Tube: AL6

Population	#Events	%Parent	%Total
All Events	64,523	####	100.0
P1	53,725	83.3	83.3
P2	46,973	87.4	72.8
P3	44,997	95.8	69.7
P4	12,091	26.9	18.7
P6	0	0.0	0.0
P5	23,365	51.9	36.2
P7	319	1.4	0.5
P8	395	0.9	0.6
P9	33,991	75.5	52.7
P10	369	1.1	0.6

Fig. S3c Gating strategy of B/T/Myeloid cell in PB in flow cytometry analysis.

1 month (3rd Tx) DR

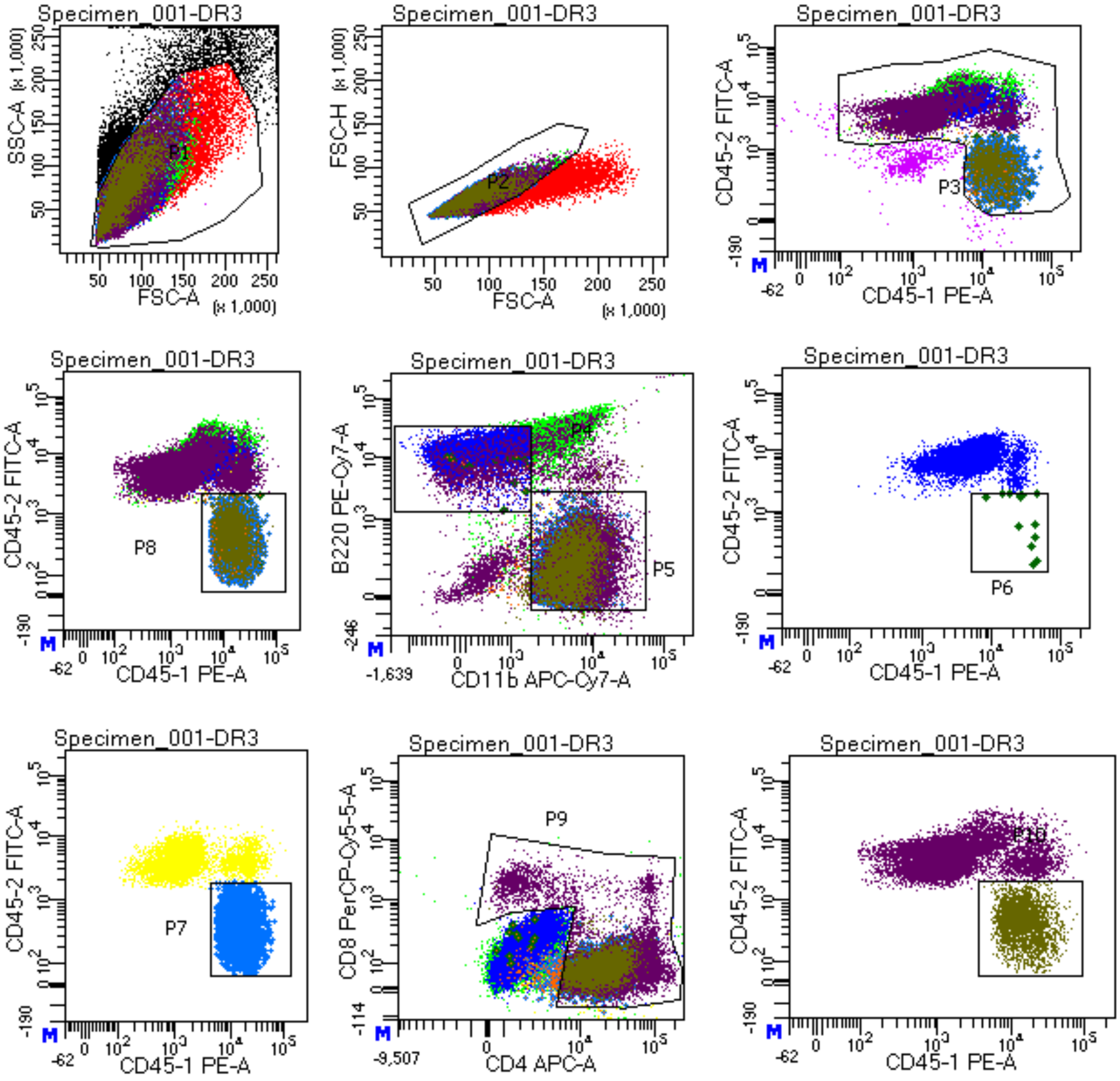

Tube: DR3

Population	#Events	%Parent	%Total
All Events	45,570	####	100.0
P1	38,576	84.7	84.7
P2	33,615	87.1	73.8
P3	32,201	95.8	70.7
P4	11,239	34.9	24.7
P6	13	0.1	0.0
P5	11,305	35.1	24.8
P7	3,105	27.5	6.8
P8	3,425	10.6	7.5
P9	14,869	46.2	32.6
P10	3,152	21.2	6.9

Fig. S3d Gating strategy of B/T/Myeloid cell in PB in flow cytometry analysis.

1 month (3rd Tx) AL

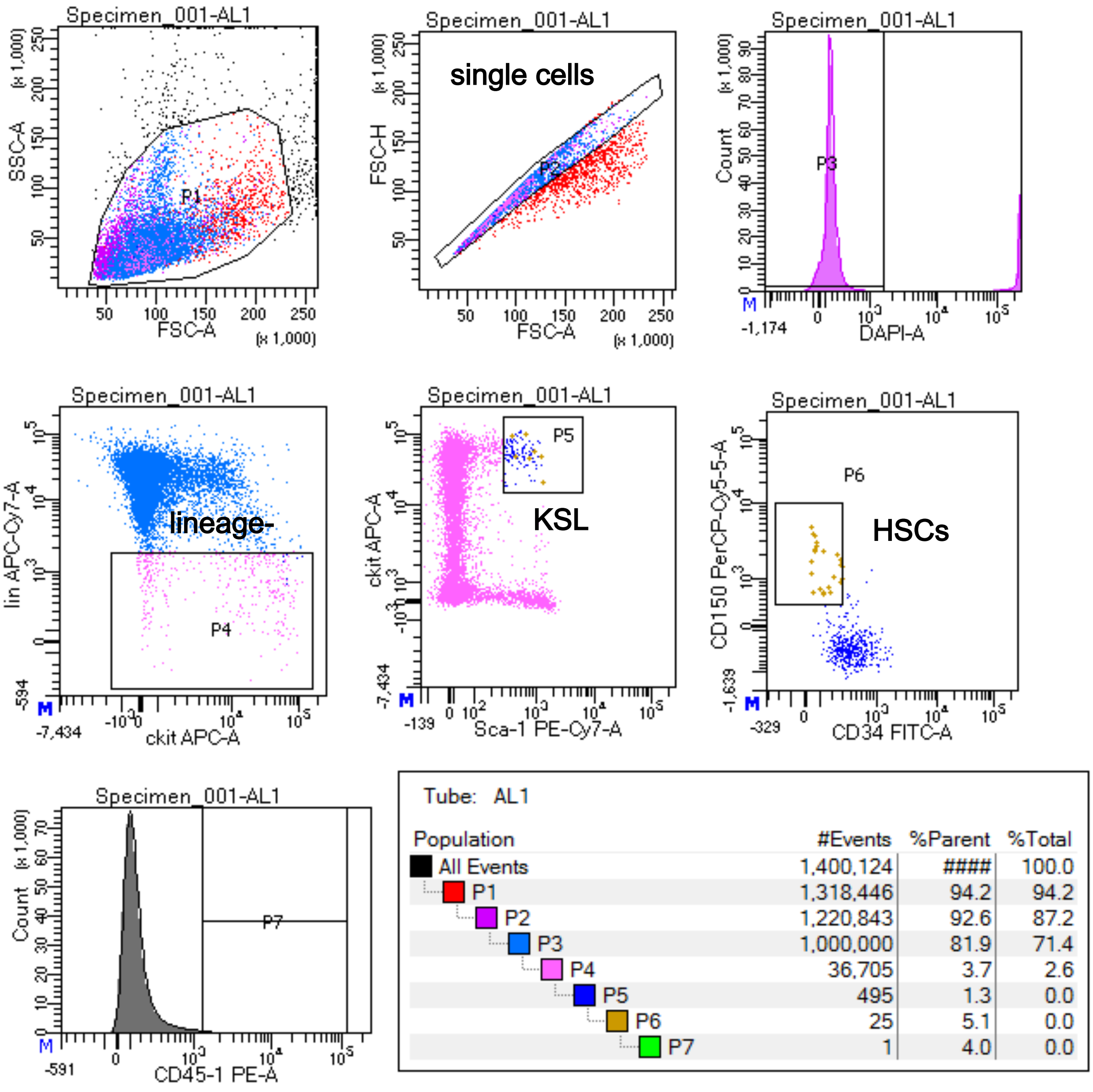

Tube: AL1

Population	#Events	%Parent	%Total
All Events	1,400,124	####	100.0
P1	1,318,446	94.2	94.2
P2	1,220,843	92.6	87.2
P3	1,000,000	81.9	71.4
P4	36,705	3.7	2.6
P5	495	1.3	0.0
P6	25	5.1	0.0
P7	1	4.0	0.0

Fig. S3e Gating strategy of HSCs in flow cytometry analysis.

1 month (3rd Tx) DR

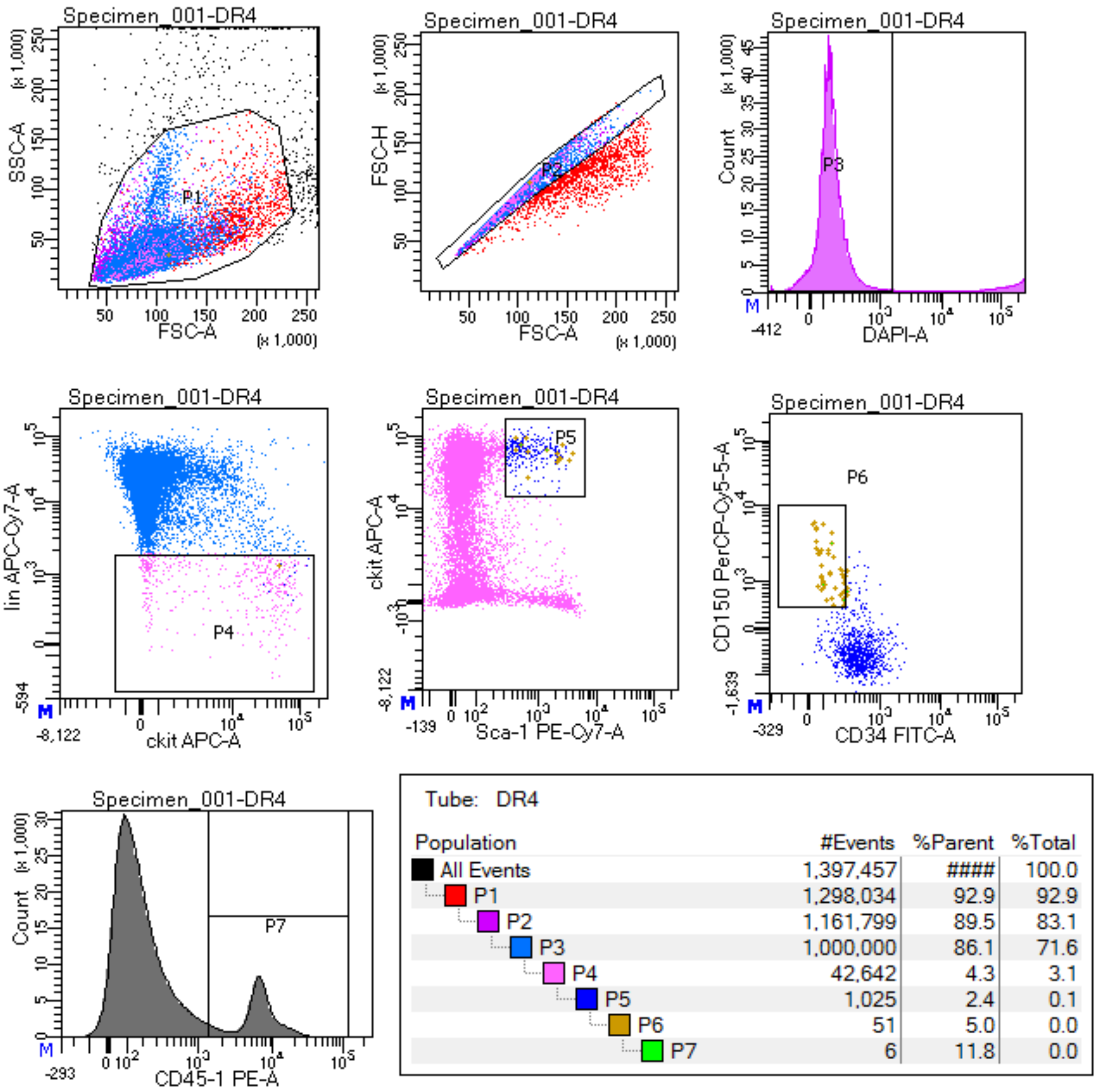

Population	#Events	%Parent	%Total
All Events	1,397,457	####	100.0
P1	1,298,034	92.9	92.9
P2	1,161,799	89.5	83.1
P3	1,000,000	86.1	71.6
P4	42,642	4.3	3.1
P5	1,025	2.4	0.1
P6	51	5.0	0.0
P7	6	11.8	0.0

Fig. S3f Gating strategy of HSCs in flow cytometry analysis.

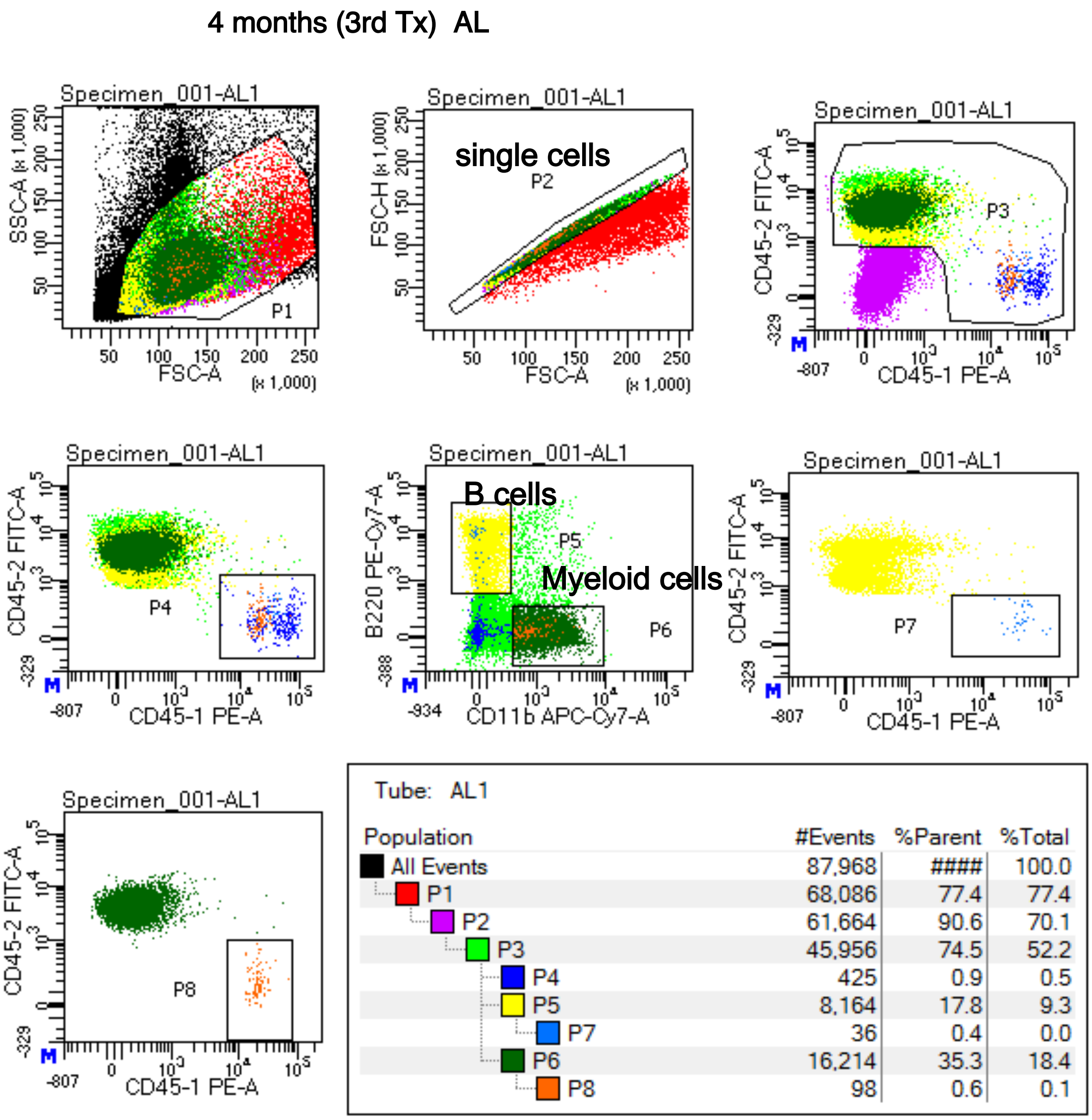

Population	#Events	%Parent	%Total
All Events	87,968	####	100.0
P1	68,086	77.4	77.4
P2	61,664	90.6	70.1
P3	45,956	74.5	52.2
P4	425	0.9	0.5
P5	8,164	17.8	9.3
P7	36	0.4	0.0
P6	16,214	35.3	18.4
P8	98	0.6	0.1

Fig. S4a Gating strategy of B/Myeloid cell in BM in flow cytometry analysis.

4 months (3rd Tx) DR

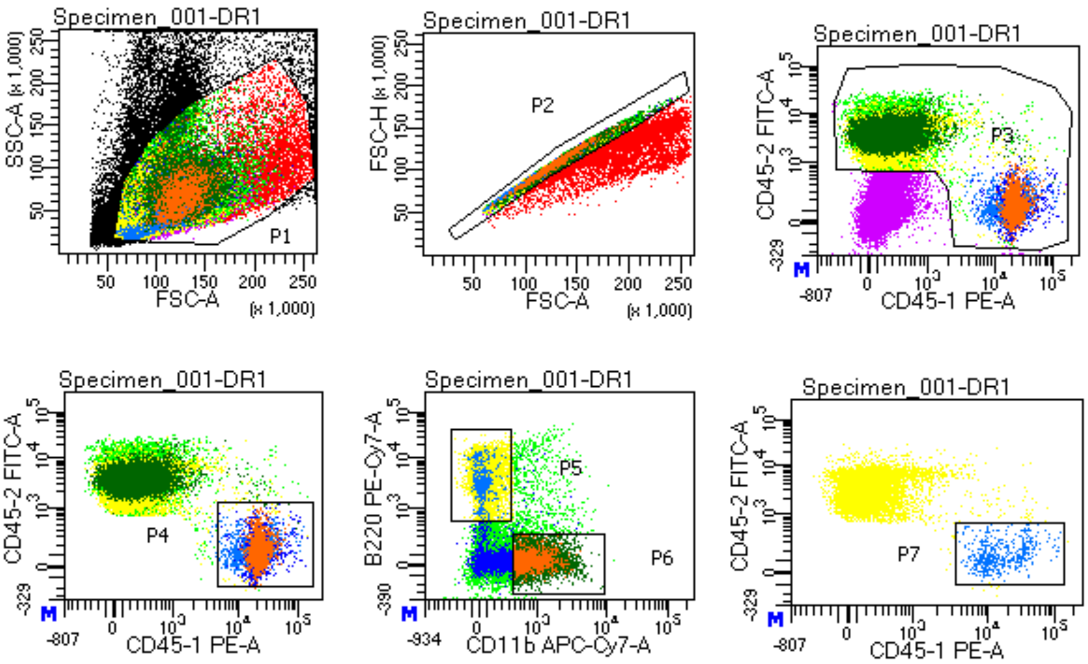

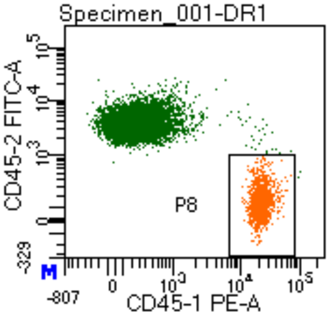

Tube: DR1

Population	#Events	%Parent	%Total
All Events	85,593	####	100.0
P1	66,654	77.9	77.9
P2	61,025	91.6	71.3
P3	48,132	78.9	56.2
P4	3,680	7.6	4.3
P5	8,239	17.1	9.6
P7	507	6.2	0.6
P6	18,124	37.7	21.2
P8	1,445	8.0	1.7

Fig. S4b Gating strategy of B/Myeloid cell in BM in flow cytometry analysis.

4 months (3rd Tx) AL

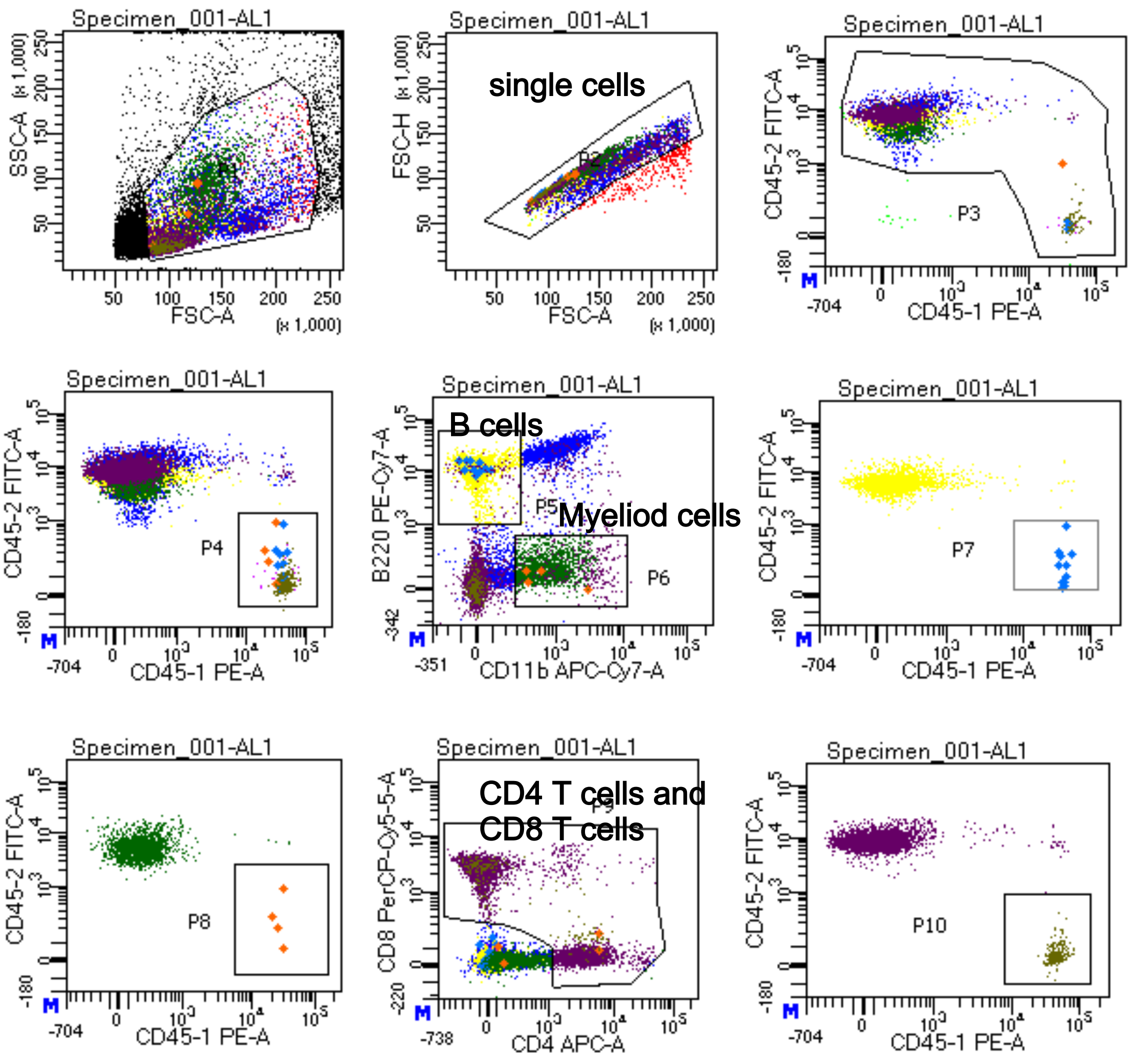

Tube: AL1

Population	#Events	%Parent	%Total
All Events	24,035	####	100.0
P1	11,763	48.9	48.9
P2	11,444	97.3	47.6
P3	11,392	99.5	47.4
P4	244	2.1	1.0
P5	2,523	22.1	10.5
P7	11	0.4	0.0
P6	1,760	15.4	7.3
P8	4	0.2	0.0
P9	4,566	40.1	19.0
P10	206	4.5	0.9

Fig. S4c Gating strategy of B/T/Myeloid cell in PB in flow cytometry analysis.

4 months (3rd Tx) DR

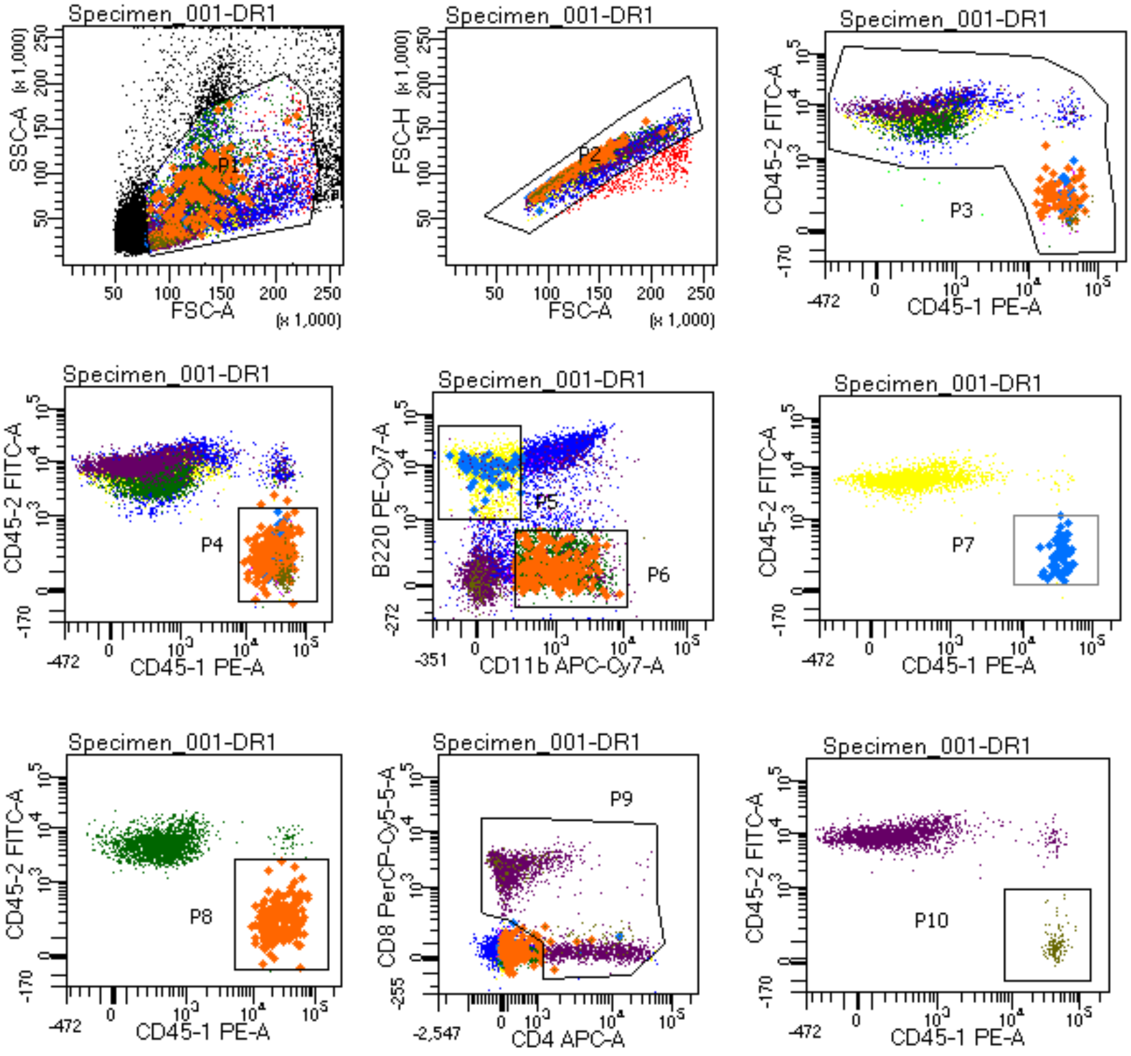

Tube: DR1

Population	#Events	%Parent	%Total
All Events	24,716	####	100.0
P1	11,901	48.2	48.2
P2	11,524	96.8	46.6
P3	11,505	99.8	46.5
P4	493	4.3	2.0
P5	2,534	22.0	10.3
P7	70	2.8	0.3
P6	2,853	24.8	11.5
P8	178	6.2	0.7
P9	2,398	20.8	9.7
P10	152	6.3	0.6

Fig. S4d Gating strategy of B/T/Myeloid cell in PB in flow cytometry analysis.

4 months (3rd Tx) AL

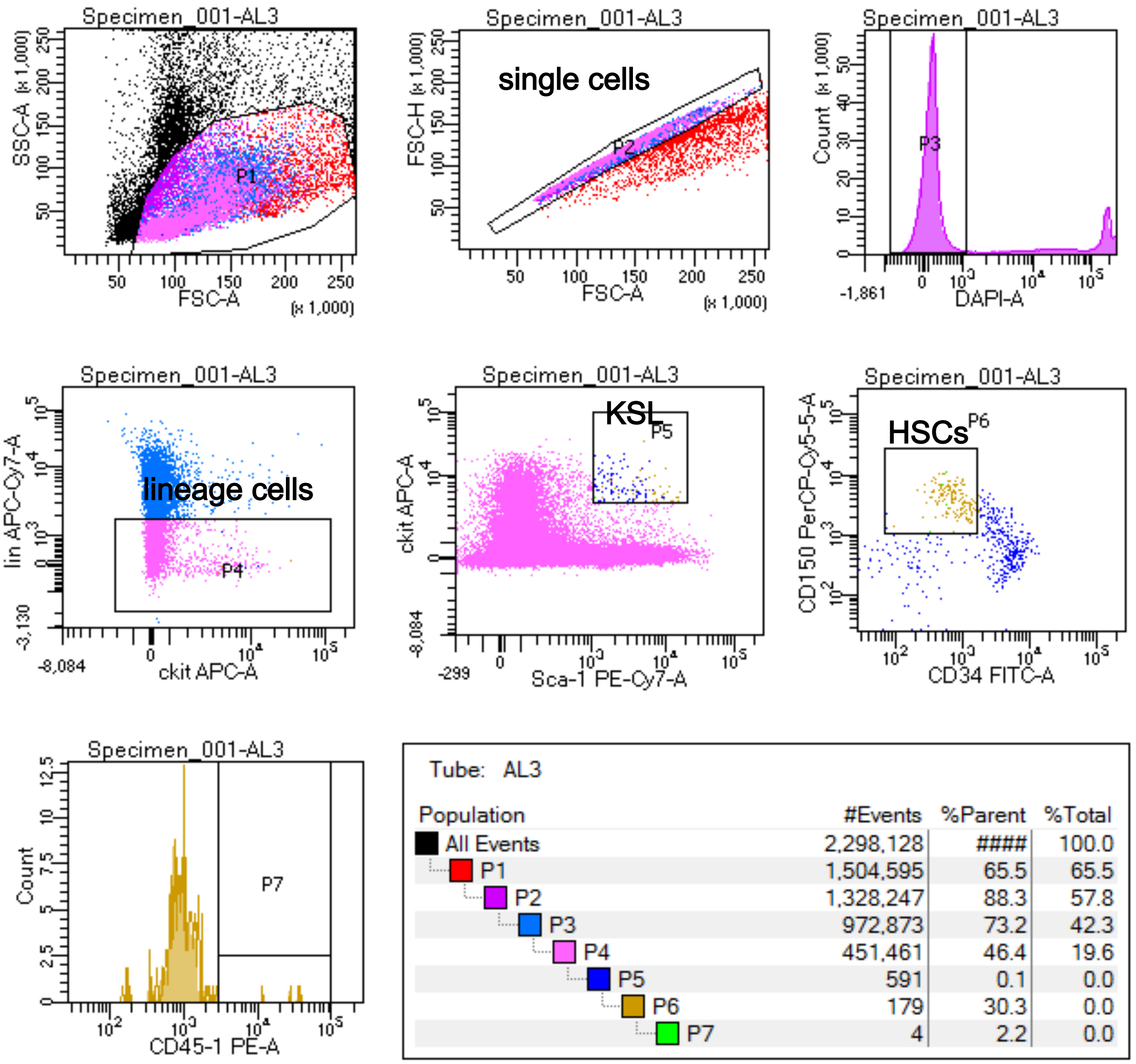

Tube: AL3

Population	#Events	%Parent	%Total
All Events	2,298,128	####	100.0
P1	1,504,595	65.5	65.5
P2	1,328,247	88.3	57.8
P3	972,873	73.2	42.3
P4	451,461	46.4	19.6
P5	591	0.1	0.0
P6	179	30.3	0.0
P7	4	2.2	0.0

Fig. S4e Gating strategy of HSC in flow cytometry analysis.

4 months (3rd Tx) DR

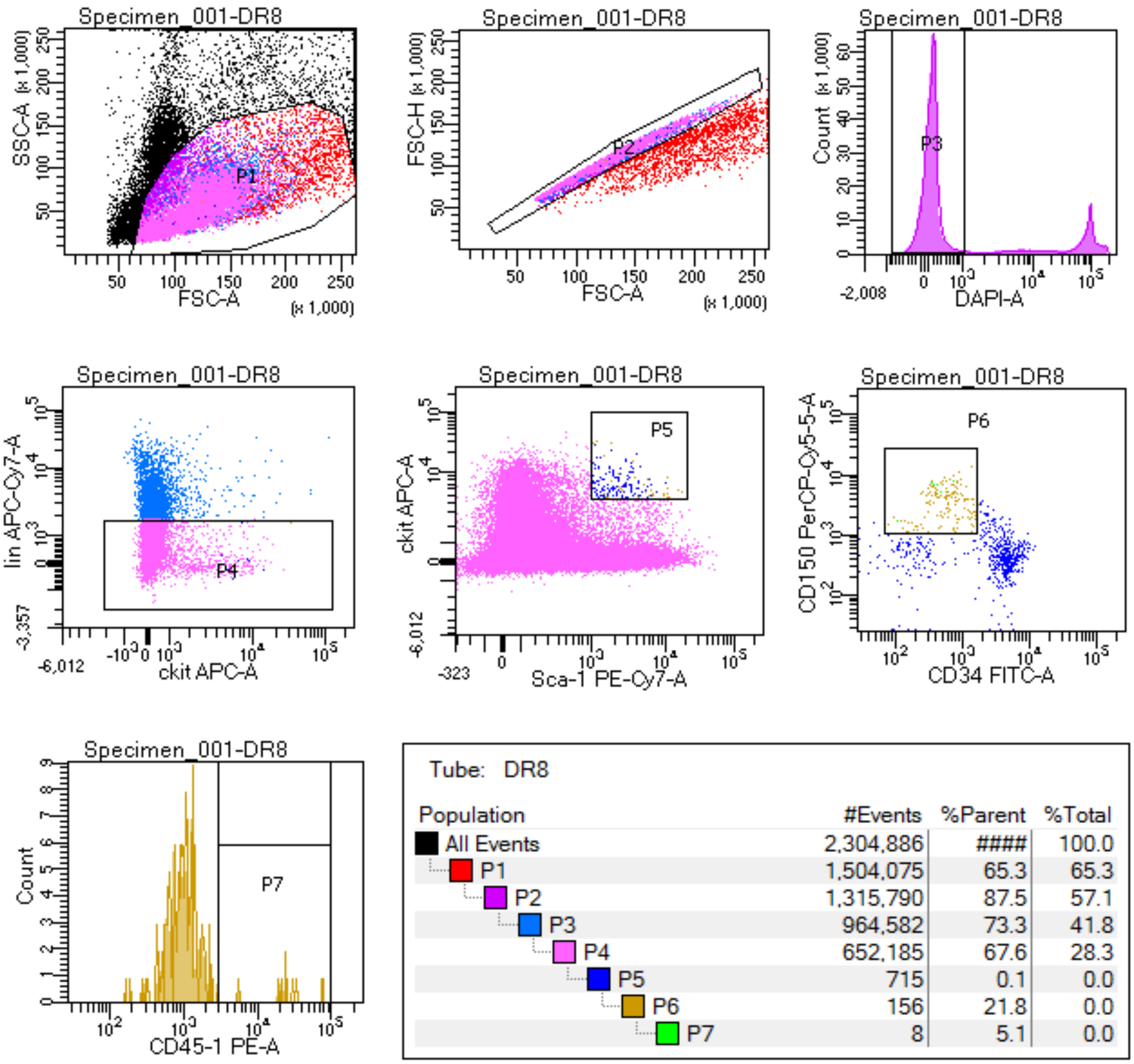

Tube: DR8

Population	#Events	%Parent	%Total
All Events	2,304,886	####	100.0
P1	1,504,075	65.3	65.3
P2	1,315,790	87.5	57.1
P3	964,582	73.3	41.8
P4	652,185	67.6	28.3
P5	715	0.1	0.0
P6	156	21.8	0.0
P7	8	5.1	0.0

Fig. S4f Gating strategy of HSC in flow cytometry analysis.

3w AL

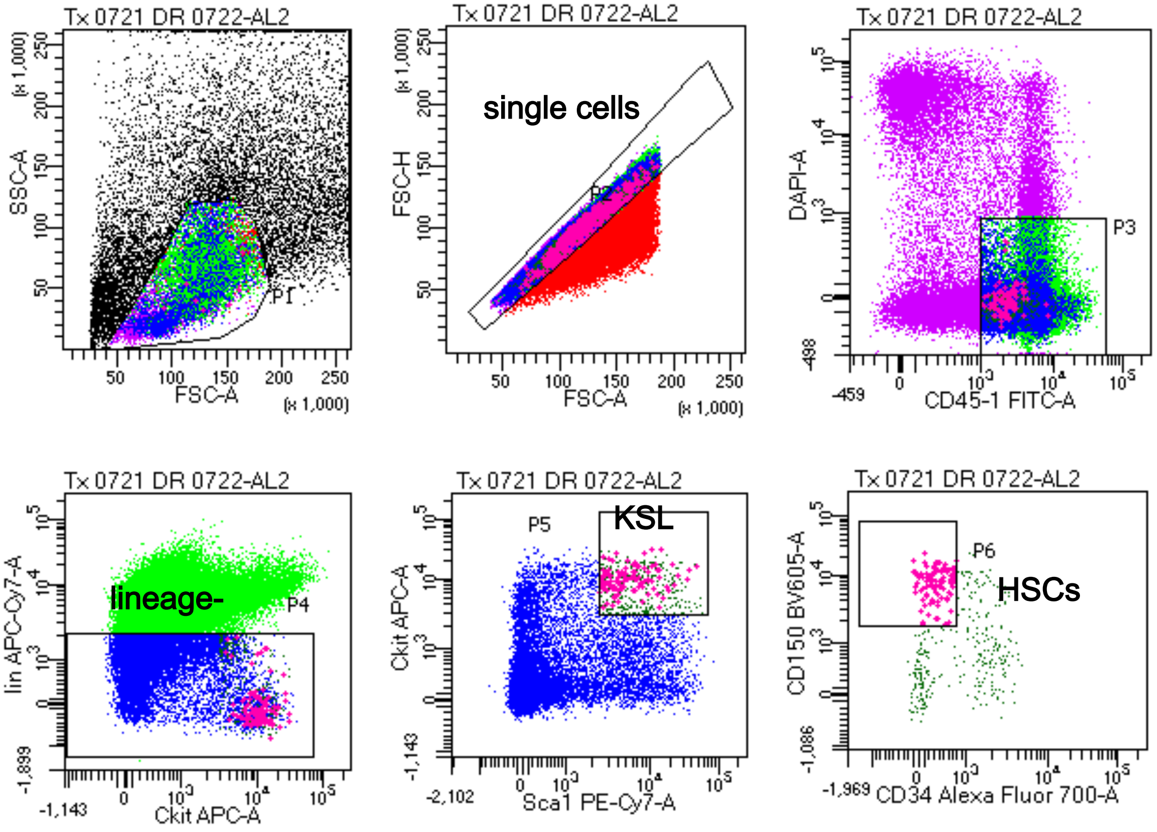

Tube: AL2

Population	#Events	%Parent	%Total
All Events	2,479,176	####	100.0
P1	1,351,037	54.5	54.5
P2	1,252,130	92.7	50.5
P3	1,054,678	84.2	42.5
P4	207,622	19.7	8.4
P5	4,261	2.1	0.2
P6	1,016	23.8	0.0

Fig. S5a Gating strategy of HSC in flow cytometry analysis.

3w DR

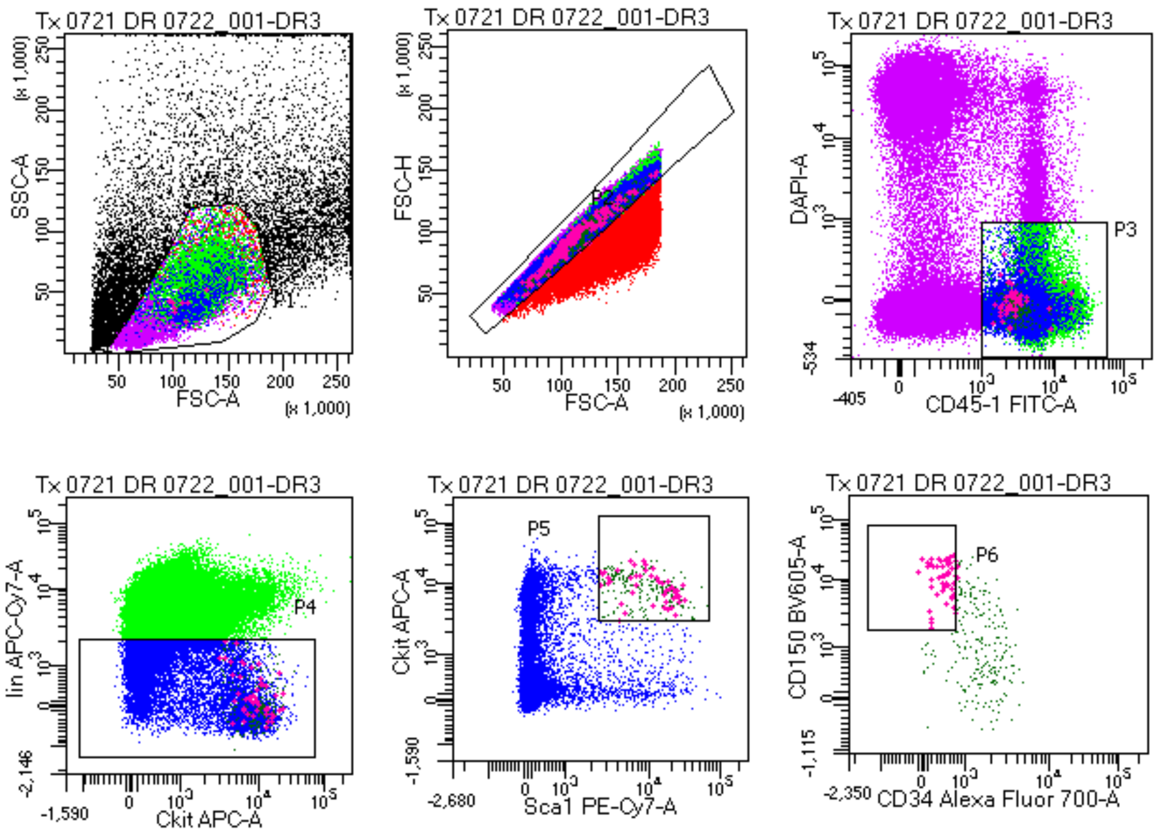

Tube: DR3

Population	#Events	%Parent	%Total
All Events	2,732,126	####	100.0
P1	1,610,029	58.9	58.9
P2	1,484,751	92.2	54.3
P3	1,002,056	67.5	36.7
P4	122,288	12.2	4.5
P5	2,416	2.0	0.1
P6	477	19.7	0.0

Fig. S5b Gating strategy of HSC in flow cytometry analysis.

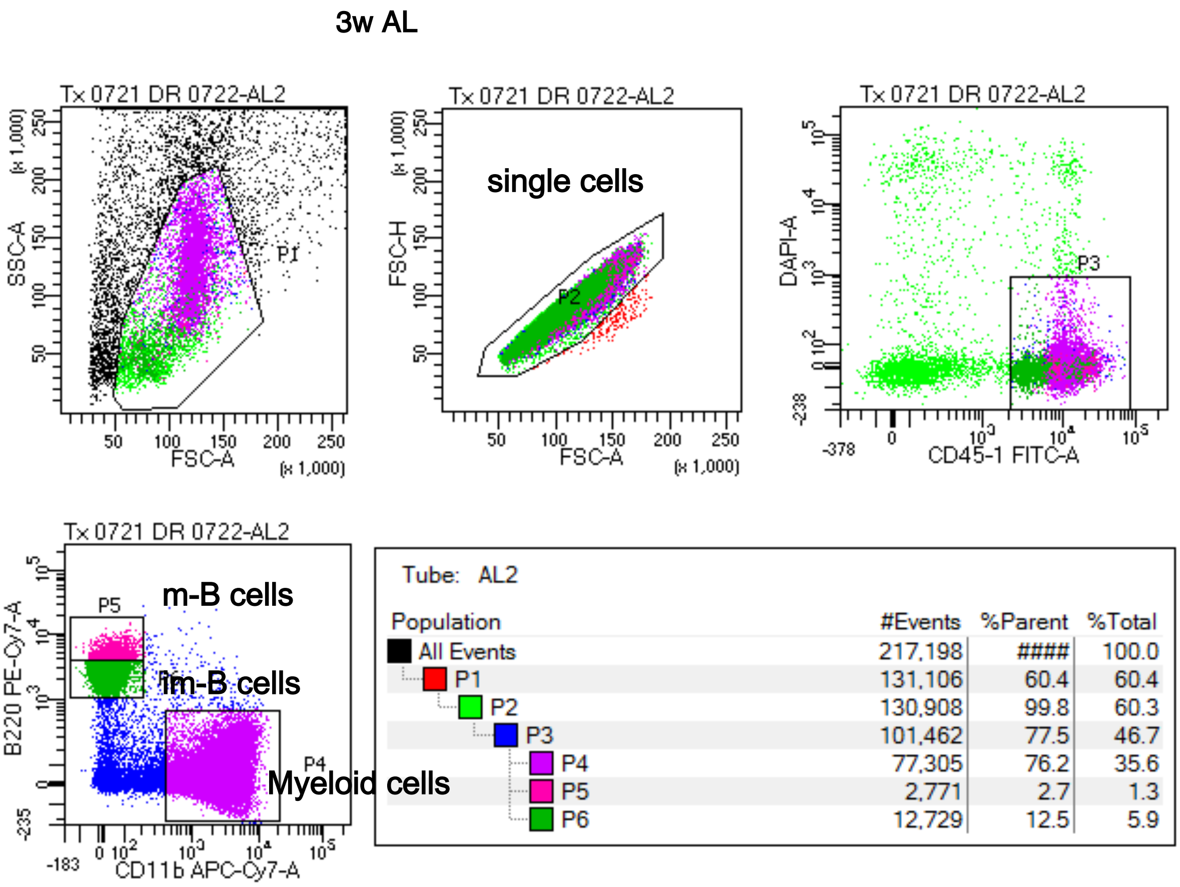

Tube: AL2

Population	#Events	%Parent	%Total
All Events	217,198	####	100.0
P1	131,106	60.4	60.4
P2	130,908	99.8	60.3
P3	101,462	77.5	46.7
P4	77,305	76.2	35.6
P5	2,771	2.7	1.3
P6	12,729	12.5	5.9

Fig. S5c Gating strategy of B/Myeloid cell in BM in flow cytometry analysis.

3w DR

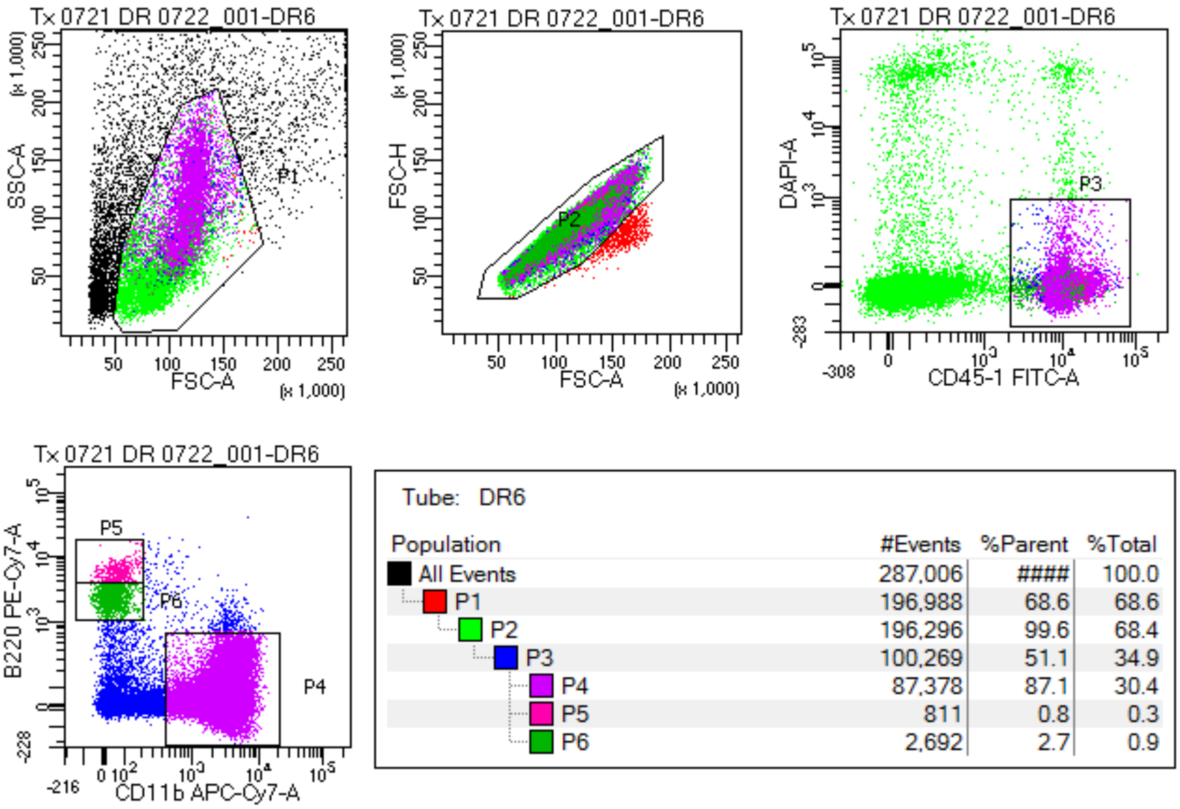

Population	#Events	%Parent	%Total
All Events	287,006	####	100.0
P1	196,988	68.6	68.6
P2	196,296	99.6	68.4
P3	100,269	51.1	34.9
P4	87,378	87.1	30.4
P5	811	0.8	0.3
P6	2,692	2.7	0.9

Fig. S5d Gating strategy of B/Myeloid cell in BM in flow cytometry analysis.

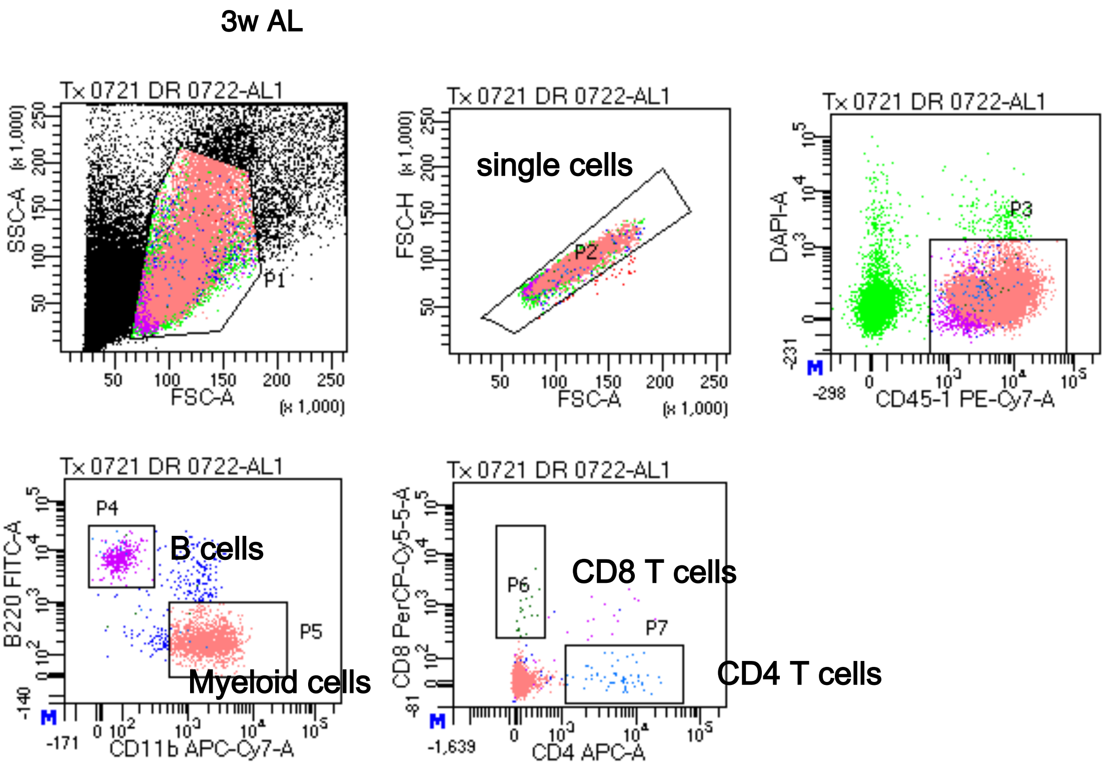

Tube: AL1

Population	#Events	%Parent	%Total
All Events	45,589	####	100.0
P1	18,604	40.8	40.8
P2	18,519	99.5	40.6
P3	11,781	63.6	25.8
P4	1,560	13.2	3.4
P5	8,759	74.3	19.2
P6	20	0.2	0.0
P7	57	0.5	0.1

Fig. S5e Gating strategy of B/T/Myeloid cell in PB in flow cytometry analysis.

3w DR

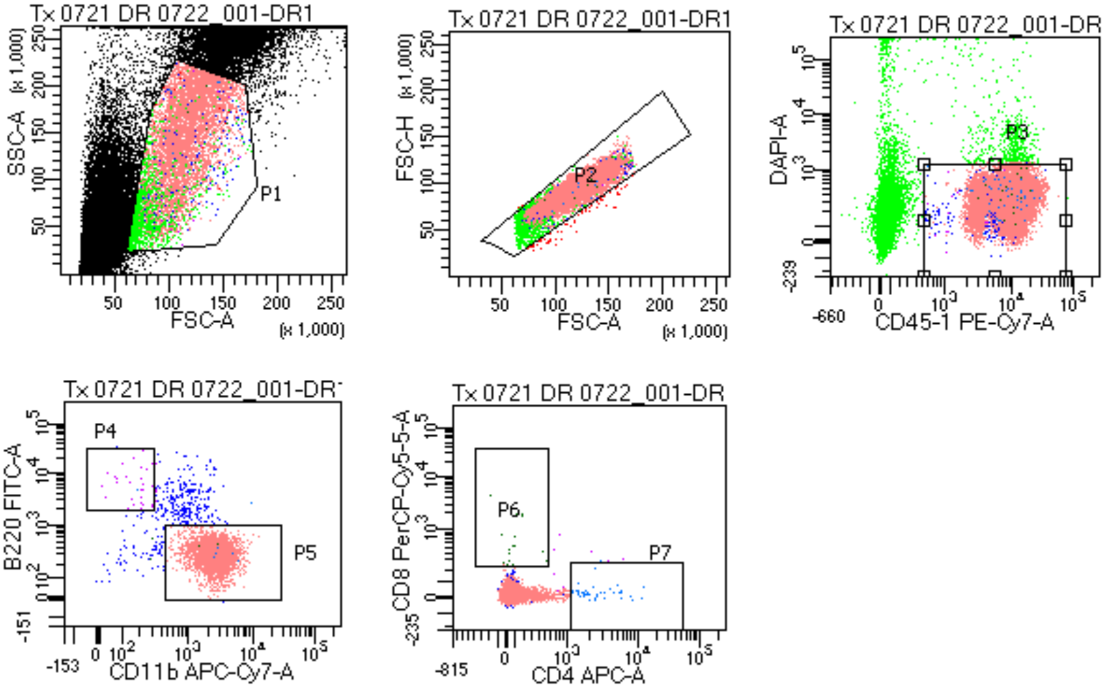

Tube: DR1

Population	#Events	%Parent	%Total
All Events	197,651	####	100.0
P1	27,994	14.2	14.2
P2	27,726	99.0	14.0
P3	20,050	72.3	10.1
P4	178	0.9	0.1
P5	18,356	91.6	9.3
P6	16	0.1	0.0
P7	43	0.2	0.0

Fig. S5f Gating strategy of B/T/Myeloid cell in PB in flow cytometry analysis.
